# Biochemical and cellular insights into the Baz2B protein, a non-catalytic subunit of the chromatin remodeling complex

**DOI:** 10.1093/nar/gkad1096

**Published:** 2023-11-24

**Authors:** Matthias Breindl, Dominika Spitzer, Rūta Gerasimaitė, Visvaldas Kairys, Thomas Schubert, Ramona Henfling, Uwe Schwartz, Gražvydas Lukinavičius, Laura Manelytė

**Affiliations:** Biochemistry III, University of Regensburg, Regensburg DE-93053, Germany; Biochemistry III, University of Regensburg, Regensburg DE-93053, Germany; Chromatin Labeling and Imaging Group, Department of NanoBiophotonics, Max Planck Institute for Multidisciplinary Sciences, Am Fassberg 11, DE-37077 Göttingen, Germany; Institute of Biotechnology, Life Sciences Center, Vilnius University, Vilnius LT-10257, Lithuania; 2bind GmbH, DE-93053 Regensburg, Germany; Biochemistry III, University of Regensburg, Regensburg DE-93053, Germany; NGS Analysis Center, University of Regensburg, Regensburg DE-93053, Germany; Chromatin Labeling and Imaging Group, Department of NanoBiophotonics, Max Planck Institute for Multidisciplinary Sciences, Am Fassberg 11, DE-37077 Göttingen, Germany; Biochemistry III, University of Regensburg, Regensburg DE-93053, Germany

## Abstract

Baz2B is a regulatory subunit of the ATP-dependent chromatin remodeling complexes BRF1 and BRF5, which control access to DNA during DNA-templated processes. Baz2B has been implicated in several diseases and also in unhealthy ageing, however limited information is available on the domains and cellular roles of Baz2B. To gain more insight into the Baz2B function, we biochemically characterized the TAM (Tip5/ARBP/MBD) domain with the auxiliary AT-hook motifs and the bromodomain (BRD). We observed alterations in histone code recognition in bromodomains carrying cancer-associated point mutations, suggesting their potential involvement in disease. Furthermore, the depletion of Baz2B in the Hap1 cell line resulted in altered cell morphology, reduced colony formation and perturbed transcriptional profiles. Despite that, super-resolution microscopy images revealed no changes in the overall chromatin structure in the absence of Baz2B. These findings provide insights into the biological function of Baz2B.

## Graphical Abstract

**Graphical Abstract:**
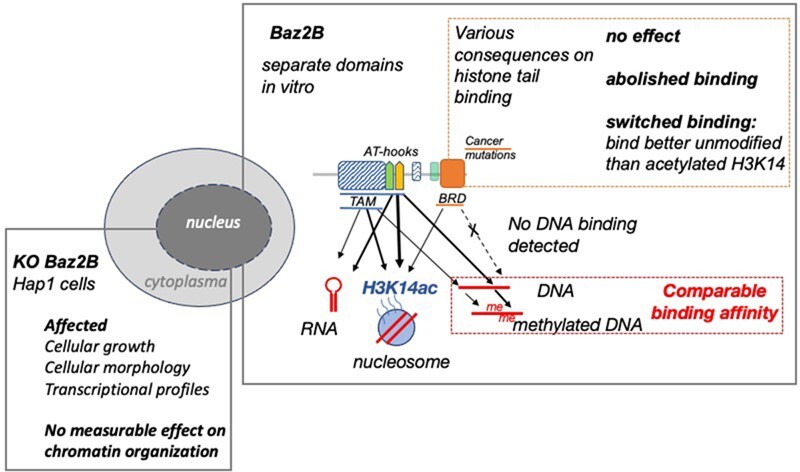

## Introduction

Nucleosomes are the key structures in eukaryotic genomes that package DNA and regulate DNA accessibility, influencing DNA-templated processes like transcription, replication, and repair (1). ATP-dependent chromatin remodeling enzymes are essential for determining nucleosome organization and positioning (2,3). These enzymes consist of catalytic subunits grouped into four families. Among the families, the ISWI chromatin remodelers include Snf2H and Snf2L proteins. These proteins contain an ATPase domain surrounded by regulatory regions AutoN and NegC, an APB motif, and a C-terminal domain known as HSS (SANT, SLIDE and HAND domains) (4–10). While ISWI family members are capable of repositioning nucleosomes independently *in vitro*, in the cellular contexts, they interact with seven regulatory subunits, mostly belonging to the Baz family, resulting in the formation of 14 distinct chromatin remodeling complexes, each with specific functions (5,11).

**Figure 1.**
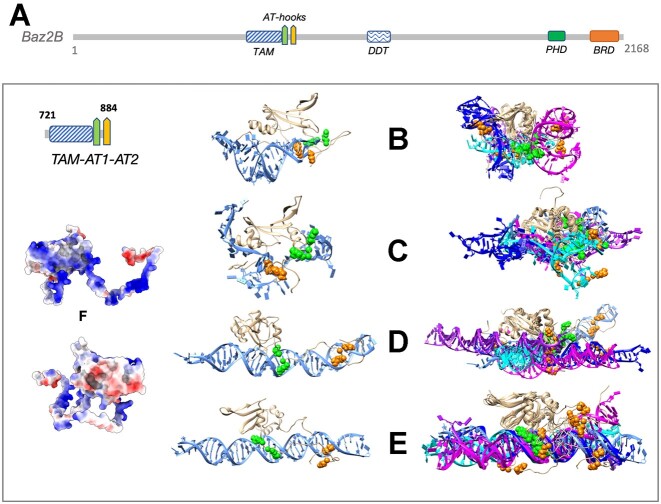
Molecular dynamics (MD) simulations of nucleic acids bound to the TAM^Baz2B^ domain with adjacent AT-hooks. The figure shows 300 ns snapshots obtained at the end of MD simulations of nucleic acids bound to the TAM^Baz2B^ domain with adjacent AT-hooks. The left side panels display the conformation representing the best protein-nucleic acid binding score, while the right side panels exhibit superposed trajectories from five independent simulations. (**A**) Domain organization of human Baz2B. TAM, Tip5/ARBP/MBD; DDT, DNA-binding homeobox and different transcription factors; PHD, plant homeodomain; BRD, bromodomain. (**B**) Snapshot of TERRA RNA interacting with the TAM-AT1-AT2^Baz2B^. (**C**) Snapshot of single-stranded DNA in a complex with the TAM-AT1-AT2^Baz2B^. (**D**) Snapshot of unconstrained DNA. (**E**) Snapshot of DNA where AT-hook1 was already bound to DNA at the beginning of the simulation, in complex with the TAM-AT1-AT2^Baz2B^. The protein is depicted in light brown, and the nucleic acids are colored in varying blueish hues to differentiate between different simulation replicas. AT-hook1 and AT-hook2 are represented as green and orange spheres, respectively, with only two residues shown for each AT-hook. (**F**) The electrostatic surface of the TAM^Baz2B^ with the adjacent AT-hooks. Solvent accessible surface colored by Coulobic potential of Baz2B TAM domain with N- and C-terminal unstructured loops that include AT-hooks. Two views of the same protein highlighting the two surfaces of the protein that have opposite electrostatic charges are shown. The surface shown on the left is much more inclined to bind nucleic acids.

In humans, two homologs of the Baz2 family, Baz2A (also known as Tip5) and the less characterized Baz2B, exist (11,12). These large proteins, with approximately ∼30% sequence identity, contain functional domains such as TAM (Tip5/ARBP/MBD), DDT (DNA binding homeobox and different transcription factors), PHD (plant homeodomain), and BRD (bromodomain) (13,14) (Figure 1A). The TAM domain shares sequence similarity with the methyl-binding domain (MBD) found in mammals, including MeCP2, MBD1-6, and SETDB1/2 (Supplementary Figure S1A, B) (12,15). While the MBDs in MeCP2 and MBD1-3 recognize methylated DNA, the MBDs of MBD5 and MBD6 are involved in protein-protein interactions (16). In contrast, the TAM domain in Baz2A has been shown to recognize distinct structural features of non-coding RNAs, such as double-helical regions in stem-loop structures (ribosomal DNA promoter RNA (pRNA)), rather than interpreting methylation marks on DNA. Although the RNA and DNA binding sites on the TAM domain of Baz2A are distinct (12,15), the existence of a ternary complex is yet to be demonstrated.

An AT-hook motifs, present in the C-terminal part of the TAM domain of Baz2A or of the MBD domain of MeCP2, synergize with the TAM or MBD domains to enhance DNA or RNA binding efficiency (12,17,18). Classical AT-hook motifs are DNA-interacting peptides containing a GRP tripeptide motif flanked by basic amino acids, which bind to the minor or major groove of AT-rich DNA sequences (19,20). Baz2A contains four canonical AT-hook motifs, two of which are located at the C-terminal part of its TAM domain (21). In Baz2B, the motifs RRGRP (potentially AT-hook 1) and RKGRP (potentially AT-hook 2) at the C-terminus of the TAM domain (Supplementary Figure S1B) remain uncharacterized and are not annotated as AT-hook motifs in the InterPro database.

The C-terminal bromodomain (BRD) of BAZ2B is the most extensively studied functional domain within the protein (22–24). Structurally, the BRD consists of four α-helices (αA, αB, αC, and αZ) with the acetylated lysine (Kac) binding cavity formed by the featured loops ZA and BC (25). The BRD domain of BAZ2B primarily recognizes acetylated Lys residues on the N-terminal tails of histones H3 and H4 (23). Interestingly, the small (S-HDAg) protein of hepatitis delta virus (HDV) can mimic histone H3 to interact with the BRD domain of BAZ2B, enabling the virus to hijack host chromatin remodelers for its RNA genome replication (26). While the BRDs of other proteins like transcription intermediary factor (TIF1α), Brg, and Brm have been reported to interact with DNA and nucleosomes, the DNA-binding activity of the Baz2B BRD remains unproven (27,28). BRD-containing proteins are often deregulated in cancer, and mutations in the BRDs themselves have been identified in a variety of cancers. The development of BRD inhibitors, including those targeting Baz2B, is now an area of intense research (22,29–35).

Baz2A and Baz2B exhibit distinct nuclear roles despite their similarities. Baz2A forms a NoRC chromatin remodeling complex with Snf2H, repositioning nucleosomes at ribosomal DNA (rDNA) promoter regions and acting as a chromatin-specific repressor of rDNA transcription by recruiting histone modifying and DNA methylating activities (36,37). Additionally, Baz2A contributes to the formation of perinuclear heterochromatin, which contains centric and pericentric repeats, thereby maintaining genome stability (38,39). In contrast, Baz2B is implicated in polymerase II activity and repression of mitochondrial genes (40). Baz2B also has been described as a master regulator in the reprogramming of human haematopoietic progenitors and has been associated with viral infections, neurodevelopment, autism spectrum disorders, cancer, and unhealthy ageing (26,40–45).

In this study, we investigate the TAM domain of Baz2B, demonstrating its interaction with single- and double-stranded DNA, nucleosomes and RNA, rather than interpreting DNA methylation marks. We further explore the presence of AT-hook motifs (AT1 and AT2) in Baz2B, which synergistically interact with the TAM domain to bind nucleic acids. Molecular dynamics simulations reveal the electrostatic interactions of the TAM domain with nucleic acids. Additionally, we show that the BRD of Baz2B does not interact with DNA, while the BRD of Brg1 exhibits DNA and triplex DNA binding activity. Furthermore, we examine the impact of cancer-associated point mutations in the Baz2B BRD, which destabilize the bromodomain, induce conformational changes, and lead to the loss or even a switch in acetylated H3 peptide binding affinity. Moreover, we investigate the effects of Baz2B depletion in Hap1 cells, uncovering decreased cell growth, morphological and transcriptional changes, while nuclear and chromatin organization remain unchanged.

## Materials and methods

### Molecular dynamics simulations

The structure of the TAM^Baz2B^ domain with adjacent AT-hooks (residues 721–884) was obtained from the AlphaFold database (46). The RMSD value between the predicted from AlphaFold and the X-ray (PDB: 7WIN) structures, with Cα atoms superimposed, is 0.78 Å (Supplementary Figure S2).

Additionally, in the X-ray structure, TAM^Baz2B^ is observed as a dimer (47). However, as suggested by the authors, this dimeric arrangement may be attributed to crystallization artefacts (60). Therefore, we performed simulations specifically focusing on the monomeric domain.

The initial 3D structure of the dsDNA (rDNA En) was constructed using Chimera, v. 1.16 (48). The initial conformation of the ssDNA (FLP015) was chosen to be helical and was also constructed using Chimera; during the MD simulation it adopted a disordered conformation due to its high flexibility when bound to the protein. The folding of the TERRA RNA was predicted by the Mfold web server (49) and its 3D structure was built by RNAserver (50). The nucleic acids were placed at 5 different arbitrary positions around the protein in the hope of at least getting a glimpse of the behaviour of the two-component system, where a wide range of conformations are possible. To study the binding of the AT-hook to En dsDNA, part of the C-terminal tail around the first AT-hook used the geometry of the analogous region of the HMG-I(Y) protein in complex with DNA (PDB ID: 2EZD) (51). Five independent molecular dynamics (MD) simulations were performed for each system using GROMACS v. 2020.4 (52). The protein and the nucleic acids were modelled using the CHARMM36m (53) and CHARMM36 (54) force fields, respectively. The protein-nucleic acid complex was placed in a dodecahedral periodic simulation box with a minimum distance of 10 Å between the solute and the box boundary. The box was then filled with CHARMM-modified TIP3P water molecules (55) and a sufficient number of Na^+^ and Cl^−^ ions to produce a salt concentration of 0.15 M and maintain the neutrality of the system. The assembled system was first subjected to steepest descent minimisation until the maximum force on any atom reached 1000 kJ mol^–1^nm^–1^. It was then subjected to 0.1 ns *NVT* equilibration using 2 fs time steps at a temperature of 300 K, with positional constraints on heavy protein and nucleic acid atoms. Production runs were performed using 2 fs time steps under *NPT* conditions at 300 K and 1 bar pressure. Temperature coupling for both *NVT* and *NPT* steps was performed using a V-rescaling algorithm with a time constant of *τ****_T_***= 0.1 ps, using nucleic acid and protein atoms as two coupled groups relative to the rest of the system. Pressure coupling for the *NPT* run was implemented using the Parrinello-Rahman algorithm with a time constant of *τ****_p_***= 2 ps and an isothermal compressibility for water of 4.5 × 10^–5^ bar^–1^. The run was performed according to the Verlet cutoff scheme. To calculate the electrostatic interactions, the Particle Mesh Ewald (PME) algorithm was implemented with a 1.2 nm cutoff and 4th order cubic interpolation with 0.16 nm grid spacing for the Fast Fourier Transform. Van der Waals interactions were filtered based on a 1.2 nm cutoff. MD production runs were 300 ns long for each of the 5 alternative simulation replicas and MD snapshots were recorded every 0.2 ns. The inter-residue contact areas, the total protein-nucleic acid interface area and the contact area-based protein-nucleic acid interaction energy were calculated using the Voronota software (56) extension Voronota-JS. The latter interaction energy was calculated based on a modified version of the VoroMQA algorithm (57) using a reduced set of atom types provided by Knodle (58). Hydrogen bonds and salt bridge hydrogen bonds were determined using ChimeraX software, v. 1.4 (48).

### DNA plasmids and strains

DNA encoding human Baz2B (UniProt: Q9UIF8) was codon optimized for eukaryotic expression, synthesized, and cloned into the pCMV3.1 vector by Genscript to generate the pCMV3.1-Baz2B-GFP plasmid. DNA encoding human TAM^Baz2B^ (721–843 aa) and TAM-AT1-AT2^Baz2B^ (721–884 aa) was codon optimized for bacterial expression, synthesised, and cloned into pET-14b or pGEX4T3 vectors by Genscript to generate pET14b-TAM^Baz2B^, pET14b-TAM-AT1-AT2^Baz2B^, and pGEX4T3-TAM-AT1-AT2^Baz2B^, respectively. Single-point mutants of TAM-AT1-AT2^Baz2B^ and BRD^Baz2B^ were generated using modifications of the Quick-change protocol as previously described (59), using pET-14b-TAM-AT1-AT2^Baz2B^ and pNIC28-Bsa4-BAZ2B-BRD as a templates, respectively, and the mutagenesis oligodeoxynucleotides (Supplementary Data Table S1). The DNA plasmids pET28a-MBD^MeCP2^ and pGEX4T3-GST and pGEX4T3-GST-AT1-AT2^BAZ2A^ were kindly provided by Skirmantas Kriaučionis and Attila Nemeth, respectively. GST-AT^Baz2B^-hook constructs were generated by introducing stop codons into the pGEX4T3-TAM-AT1-AT2^Baz2B^ DNA plasmid as described (59). All generated plasmids used in this study were sequenced. DNA plasmids pNIC28-Bsa4-BAZ2B-BRD (Addgene #39000) and pNIC28-Bsa4-Smarca4 BRD (Addgene #39116) were a kind gift from Nicola Burgess-Brown and were used for overexpression and purification of His_6_-BRD^Baz2B^ and His_6_-BRD^Brg1^, respectively. The *E.coli* DH5α (Invitrogen) and BL21(DE3)-R3-pRARE2 (kind gift from SGC (Structural Genome Consortium)) or Rosetta pLysS strains were used for the cloning and recombinant protein expression, respectively.

### Expression and purification of recombinant proteins

Recombinant His_6_-BRD^Baz2B^, His_6_-BRD^Brg1^, His_6_-TAM^Baz2B^, His_6_-TAM-AT^Baz2B^ proteins, and the single-point mutants were purified from BL21(DE3)-R3-pRARE2 cells transformed with pNIC28-Bsa4-BRD^Baz2B^, pNIC28-Bsa4-BRD^Brg1^, pET14b-TAM^Baz2B^ and pET14b-TAM-AT1-AT2^Baz2B^, respectively.

Recombinant His-MBD^MeCP2^ was purified from Rosetta plysS. An overnight culture was used to inoculate 1 l of LB broth supplemented with 100 μg/ml ampicillin and 34 μg/ml chloramphenicol. Cultures were grown at 37°C for 16 hours until A_600_ was ∼ 0.5–0.8, induced by the addition of 0.5 mM IPTG, and incubated overnight at 18°C. The cells were harvested by centrifugation at 4°C, and the pellet was resuspended in 20 ml of lysis buffer (50 mM HEPES pH 7.5, 500 mM NaCl, 5% (v/v) glycerol, 5 mM imidazole) supplemented with 0.5 mM TCEP (ThermoFisher Scientific) and protease inhibitors (100 μM leupeptin and 100 μM PMSF). Cells were lysed on ice using a Dounce homogeniser and sonication. The soluble fraction was collected by centrifugation. The His-tagged proteins were batch-purified from the lysate. The lysate and 1ml of pre-washed Ni-NTA agarose beads (Qiagen) were incubated for 1h on a rotating wheel at 4°C. The Ni-NTA agarose beads were then washed in wash buffer (50 mM HEPES pH 7.5, 500 mM NaCl, 5% (v/v) glycerol, 30 mM imidazole) and protein was eluted by gravity flow in elution buffer (50 mM HEPES pH 7.5, 500 mM NaCl, 5% (v/v) glycerol, 250 mM imidazole). Fractions containing the protein of interest were combined, concentrated using an Amicon concentrator/ cut off 3kD (Merck) and then applied to a Sephadex 75 column (GE Healthcare) on an ÄKTA FPLC in buffer A (50 mM HEPES pH 7.5, 500 mM NaCl, 5% (v/v) glycerol). Fractions containing only the protein of interest were combined, concentrated, snap-frozen, and stored at -80°C. The GST-fusion proteins were expressed and purified as described (21).

### Determination of protein melting temperatures

Differential scanning fluorimetry (nanoDSF) was used to measure the thermal unfolding of proteins and to determine protein melting temperatures. This method is based on the intrinsic fluorescence of the amino acids tryptophan and tyrosine along a thermal unfolding ramp (60). The capillaries were filled with 30 μM protein in buffer A and placed on the sample holder of the Prometheus NT.48 instrument (NanoTemper Technologies). A temperature gradient of 1°C min^−1^ from 25 to 95°C was applied and intrinsic protein fluorescence was recorded at 330 and 350 nm. The fluorescence intensity ratio and its first derivative were calculated using the manufacturer's software (PR.ThermControl, version 2.1.2). Three independent measurements each with two technical duplicates were carried out for each condition and the mean is presented.

### Oligonucleotide substrates

All DNA and RNA oligonucleotides were purchased from Sigma-Aldrich and MWG-Biotech, respectively, and were HPLC-purified by the manufacturers. The oligonucleotides were fluorescently labelled at the 5′-end or unlabelled. The list of oligonucleotides used is given in Supplementary Data Table S2. Double-stranded DNA substrates were prepared by mixing single-stranded oligonucleotides in annealing buffer (10 mM Tris–HCl pH 7.5, 50 mM NaCl, 1 mM MgCl_2_), heating at 95°C for 2 min and then slowly cooling down to room temperature. Unlabelled oligonucleotides were present in 1.2-fold molar excess over the labelled oligonucleotides. A double-stranded 45 bp DNA substrate was prepared by annealing FLP015 and FLP016. To prepare a DNA triplex, consisting of three individual intertwined helices, 1 μM triplex-forming oligonucleotide TFO #5 and 1 μM 29 bp En3_D dsDNA were incubated in TA buffer (40 mM Tris-Ac and 15 mM MgAc, pH 7.4) supplemented with 0.05% NP-40 in a total volume of 20 μl at 37°C for 30 minutes, then cooled to 25°C at 1°C/min. DNA triplexes were stored on ice and used immediately.

### Electrophoretic mobility shift assay

RNA and DNA binding of TAM^Baz2B^, TAM-AT1-AT2^Baz2B^, and the point mutants was determined by electrophoretic mobility shift assay (EMSA). Briefly, 2 μl of diluted proteins in buffer A were incubated with 10 nM fluorescence-labelled RNA, ssDNA, or dsDNA (Supplementary Data Table S2) in EMSA buffer (20 mM HEPES pH 7.5, 30 mM KCl, 1mM EDTA, 10 mM (NH_4_)_2_SO_4_) supplemented with 1 mM dithiothreitol in a total volume of 10 μl. After 30 min at 4°C, 4 μl of 50% glycerol solution was added, the samples were placed on ice and loaded onto 6% native polyacrylamide gels in 40 mM Tris (pH 7.5), 20 mM sodium acetate, 1 mM EDTA. Gel electrophoresis was performed at 4°C under 11.4 V/cm for 50 min, after which the gels were scanned using Typhoon FLA-9500 laser scanner (GE Healthcare). Gel images were analysed using ImageJ and SigmaPlot. To analyse DNA binding of recombinant AT^Baz2B^-hooks and BRD^BRG1^ and BRD^Baz2B^, EMSAs were performed as described in (21) and (61), respectively. To test bromodomain binding to DNA, the Widom 147 bp DNA substrate was used and it was prepared as described (62).

### Microscale thermophoresis assay

Microscale thermophoresis (MST) experiments were performed on a Monolith NT.115 device (NanoTemper Technologies GmbH). The Monolith NTTM Protein Labelling Kit RED-NHS (NanoTemper Technologies GmbH) was used for protein labelling, according to the manufacturer's instructions. Samples were prepared in the MST buffer (150mM Tris–HCl, 50mM NaCl, 10 mM MgCl_2_, pH 7.6) supplemented with 0.0025% (v/v) Pluronic and 200 ng/μl BSA and 100 nM fluorescently labelled BRD^Baz2B^. For each binding analysis, a titration series was prepared with varying concentrations of the respective peptide (H3 or H3K14ac). MST measurements were performed at 25°C using 20% MST power with laser on the 30s, off 5s, and delay 25s. All experiments were conducted at least in triplicate. Thermophoresis signals were normalised to fraction bound (X) by X= [Y(c)− Min]/(Max − Min), error bars (SD) were normalized by std_norm_= std(c)/(Max − Min), and both were plotted in Microsoft Excel (21). The *K*_d_ constants between the protein and the ligands were calculated from the saturation binding curve at equilibrium (63).

To test the affinity of TAM^Baz2B^ and TAM-AT1-AT2^Baz2B^ for nucleic acids, the 5 nM fluorescently labelled RNA or DNA was incubated with different concentrations of proteins in binding buffer (20 mM HEPES pH 7.5, 30 mM KCl, 1 mM EDTA, 10 mM (NH_4_)_2_SO_4_, 0.1 μg/ml BSA, 1 mM DTT) to a final volume of 12 μl. Samples were incubated for 15 minutes in the dark on ice and loaded onto appropriate capillaries (NT.115 Standard/Premium capillaries). The Monolith NT.115^pico^ for ‘LED Power’ was set to a value between 10% and 20% so that the ‘Raw Fluorescence’ was between 5000 and 20 000 counts. Measurements were performed at 25°C with 60% MST power, with laser on for 30 s, off for 5 s and delay for 25 s. Data were analysed using MO.Affinity Analysis software. Experiments were performed at least in triplicate. Binding affinities were evaluated using non-linear regression (binding saturation, specific binding with Hill slope) with GraphPad Prism.

### Peptides and antibodies

H3K14ac (Ac-TARKSTGGK(ac)APRKQL-NH_2_) and H3 (Ac-TARKSTGGKAPRKQL-NH_2_) peptides were purchased from (alta Bioscience), and dissolved in 10 mM Tris pH 7.6 at 20 mM final concentration and stored at -80°C. Baz2B antibody (Abiocode, C2), HRP-Goat anti-rabbit IgG (H + L) (Jackson ImmunoResearch, #111–035-144), anti-tubulin (Abcam, #ab6161) HRP-Goat anti-rat IgG + IgM (H + L) (Jackson ImmunoResearch, #112–035-068) were used. Nucleolin was stained with rabbit polyclonal anti-nucleolin antibody (Abcam, #ab22758) followed by donkey anti-rabbit IgG (H + L) highly cross-adsorbed secondary antibody, Alexa Fluor™ Plus 488 (Invitrogen, #A32790). Histone modifications were stained with anti-histone H3K4ac (Active Motif, #39382), anti-histone H3K9me2 (Active Motif, #39754) or anti-histone H3K27ac (Active Motif, #39134) antibodies, followed by goat anti-rabbit IgG labelled with Abberior STAR RED (Abberior, #STRED-1002–500UG).

### Cell culture and treatment

Baz2B-KO (HZGHC000523c005, 16 bp deletion in the coding exon in Baz2B was edited by CRISPR-Cas9) and its parental Hap1 cell lines were purchased from Horizon Discovery (Cambridge, UK). Cells were cultured in IMDM (from Gibco™, serum-free medium) supplemented with l-glutamine, phenol red indicator, and 10% FCS. Cells were typically maintained at 40–90% confluence and passaged every 2–3 days. The two cell lines were treated separately.

An inducible U2OS cell line expressing BAZ2B-halo was generated by cloning the BAZ2B gene (UniProt: Q9UIF8, codon optimised by Genescript) into the pEBTet GW_Halo plasmid vector, transfecting and selecting plasmid-containing cells in McCoys 5A medium (ThermoFisher #16600082) with 10% FBS (BioSELL, #S0615) supplemented with 1 mM sodium pyruvate (Sigma, #S8636), 1% penicillin-streptomycin (Sigma #P0781) and 1 μg/ml puromycin (Sigma, #P9620) in a humidified 5% CO2 incubator at 37°C for 1–2 weeks (64,65). For imaging, the cells were grown in μ-Slide 8-well glass bottom slides (Ibidi) for 48 h and gene expression was induced with 0.1 μg/ml doxycycline for a further 24 h. The BAZ2B-halo was stained by incubation with 100 nM JF646-halo for 1 h and the cells were fixed with 4% PFA.

### Imaging

To determine overall nuclear morphology, the cells were stained with 1 μg/ml Hoechst 33342 in the growth medium for 1 h and imaged without washing on an Abberior Facility line STED microscope (Abberior Instruments GmbH) in confocal mode using the Olympus UPlanSApo 60×/1.42 oil objective.

For high-resolution imaging of chromatin structure, passage 24 Hap1 cells were stained with 100 nM 5-HMSiR-Hoechst (66) in growth medium for 1 h at 37°C and imaged at room temperature without washing. 30 000 frames were acquired at 100Hz on a Visitron spinning disc/TIRF/SMLM system (Visitron Systems GmbH, Germany) equipped with a Nikon CFI Apochromat TIRF 100 × C Oil NA 1.49 objective and a Prime 95B sCMOS camera (Teledyne Photometrics). A 640 nm laser with a power of 18 kW/cm^2^ was used for excitation. The high-resolution images were reconstructed using SVI Huygens localizer software (version 22.04.0p3) and were rendered with a pixel size of 10 nm and an FWHM of 30 nm.

### RNA extraction, reverse transcription, and quantitative PCR (qPCR)

Total RNA was extracted using the Macherey-Nagel NucleoSpin® RNA Kit (Bioanalytik). RNA concentration was measured using NanoDrop. Subsequently, cDNA was synthesised from 1 μg of total RNA using the Bio-Rad iScript™ cDNA synthesis kit according to the manufacturer's instructions. A qPCR reaction (total 20 μl) contained 0.4 μM of the respective forward and reverse primers (Supplementary Data Table S3), 4 μl of cDNA and PCR buffer 1×, 0.2 mM dNTPs (each), 1mM MgCl_2_, 0.02 U HotStarTaq (Qiagen) DNA polymerase and a 1:400 000 dilution of a 10 000× Sybr green I stock (Roche). The quantitative PCR run was programmed as follows: hold for 10 min at 95°C, cycling 35× (20 s at 95°C, 20 s at 60°C, 20 s at 72°C), melting from 72°C to 99°C, followed by a 5 s for hold for step. Data were collected using a Rotor-Gene 3000 system (Corbett Research/Qiagen) and analysed using a comparative analysis software module. All samples were run in technical triplicate and GAPDH was used for normalisation. Means and error bars were calculated in Microsoft Excel and derived from three independent biological replicates. Relative expression values for control and Baz2B-KO conditions were tested for significance using a two-sample, assuming equal variances *t*-test in Excel. *P*-values are given for sets with significant changes (*P< 0.05, **P< 0.01, ***P< 0.001).

### Clonogenic assay

Cells were seeded into 35mm plates containing 5 ml IMDM medium supplemented with 10% FCS in quintuplicate at 100, 500 and 1000 cells/plate. On day 7, the medium was aspirated and colonies were stained with 3 ml 0.01% (w/v) crystal violet for 20 minutes, afterward the plates were rinsed several times with water and air-dried. The plates were scanned and colonies were counted using clono.jar software. Experiments were performed in triplicate. Means and error bars (*N9*) were calculated using Microsoft Excel. Relative colony counts for Hap1 control and Baz2B-KO conditions were tested for significance using a two-sample, assuming equal variances *t*-test in Microsoft Excel. *P*-values are given for sets with significant changes (*P< 0.05, **P< 0.01, ***P< 0.001).

### Microarray data analysis

Microarray analysis was performed using on the Bioconductor tutorial ‘An end-to-end workflow for differential gene expression using Affymetrix microarrays’ (67). Data were imported into the R environment and further processed using the Bioconductor oligo package (68). Probe intensities were normalised using the *rma* function. Low-intensity probes were removed and only probes with intensities greater than 3.5 in at least two samples were retained for further analysis. Probe IDs were assigned to Ensembl gene IDs using the Bioconductor package AnnotationDbi (69), and probes with no or multiple assignments were removed. To identify significant changes in transcript abundance in the BAZ2B-KO condition, a linear model was fitted to control Hap1 samples (*n* = 2) and the BAZ2B-KO Hap1 samples (*n* = 2) using the Bioconductor limma package (70). After normalisation for multiple testing (FDR), 99 differentially expressed genes were detected that passed the significance threshold of 0.05. The R code for the analysis workflow is available on GitHub. Gene ontology enrichment analysis of the down- and up-regulated genes was performed using Metascape (71).

## Results

### Molecular dynamics simulation of the TAM^Baz2B^ domain and its auxiliary AT-hooks binding to nucleic acids

There is increasing structural data on how the TAM domain of Baz2A interacts with the DNA or RNA (15,72). Interestingly, only the structure of TAM^Baz2A^ was solved in the presence of DNA, demonstrating binding to the DNA backbone in a sequence-unspecific manner. However, the apostructure of TAM^Baz2B^ was resolved despite the presence of DNA in the experimental setup (47). Our sequence alignment data (Supplementary Figure S1A) indicated the potential presence of nucleic acid binding properties in the C-terminus of the TAM^Baz2B^, suggesting a similarity to AT-hook motifs (TAM-AT1-AT2^Baz2B^).

To investigate the nucleic acid binding properties of TAM^Baz2B^ further, we conducted molecular dynamics (MD) simulations of a monomeric TAM domain with C-terminal extension, potentially AT-hooks, from Baz2B (residues 742–884) in the presence of double- (dsDNA), single-stranded (ssDNA) DNA, and RNA. For these simulations, we used ribosomal DNA (rDNA) promoter and enhancer DNA sequences, as well as the readily available TERRA (Telomeric-Repeat-containing RNA) RNA structure, which are known substrates for the Baz2A protein. Five independent 300 ns MD simulations were carried out for each system. The results of the simulations showed diverse nucleic acid binding conformations of the TAM-AT1-AT2^Baz2B^ domain at the end of the 300 ns trajectories (Figure 1 B-D).

The interaction strength and interface areas between the nucleic acids and the protein varied considerably among the simulation replicates (Table 1), indicating the presence of multiple binding modes. The presence of numerous hydrogen bonds and salt bridges, especially in the case of dsDNA bound to the protein, further supported the notion that the TAM-AT1-AT2^Baz2B^ domain functions as a nucleic acid interaction module.

**Table 1.** Averaged interaction parameters between Baz2B TAM domain to the nucleic acids

Nucleic acid	Interface area (Å^2^)	Interaction score^a^	H-bonds	Salt bridges^b^
TERRA RNA	1319 ± 155	−406 ± 71	25.1 ± 6.2	10.1 ± 1.7
ssDNA FLP15	1895 ± 578	−633 ± 232	35.8 ± 11.6	13.2 ± 3.5
rDNA En (unconstrained)	1321 ± 311	−310 ± 46	85.7 ± 7.2	8.2 ± 3.9
rDNA En (bound AT-hook1)	1559 ± 208	−418 ± 71	90.1 ± 8.2	8.6 ± 3.0

The parameters relate the interaction across the protein-nucleic interface. The values were calculated for the last 2 ns of the 300 ns simulation, averaged over 5 trajectories (hence large standard deviations). ^a^Unitless interaction pseudo-energy. ^b^Number of salt-bridging hydrogen bonds. For more details and the used software, see Materials and methods.

Additionally, we conducted simulations that focused on the interaction of the AT-hooks with DNA (Figure 1E). By transferring the geometry of the AT-hook from the PDB file (PDB ID: 2EZD) to the first AT-hook (AT1) as a part of the Baz2B TAM domain, we observed that both AT-hooks, especially AT1, played significant roles in nucleic acid binding, mediated by electrostatic interactions and hydrogen bonding. Comparing the images on left sides of Figure 1D and E reveals that the best binding conformation from the unconstrained simulation (Figure 1D) is similar to the simulation with the AT-hook1 bound (Figure 1E). It is noteworthy that the AT-hook 2 (shown in orange in Figure 1) often makes contact with the major groove of dsDNA (Figures 1D, E, and Supplementary Figure S3A, B), but the simulation time is insufficient for it to wedge deeply into it, similar to AT-hook 1 in Figure 1E.

Furthermore, our findings also revealed an uneven distribution of charged residues on the surface of the TAM–AT1–AT2 domain (Figure 1F), explaining why nucleic acids predominantly bind to one side of the domain while the other side remains mostly free of contact with nucleic acids in MD simulations. The presence of a positively charged surface continuum, including AT-hook 1 and AT-hook 2, facilitated the binding of the nucleic acids.

Overall our MD simulations demonstrated that the binding of DNA and RNA to the TAM domain is primarily driven by electrostatic interactions, leading to multiple binding modes und further underscored the versatility of the TAM–AT1–AT2 domain in nucleic acid interactions.

### TAM domain and its auxiliary AT-hook motifs bind synergistically to nucleic acids

**Figure 2.**
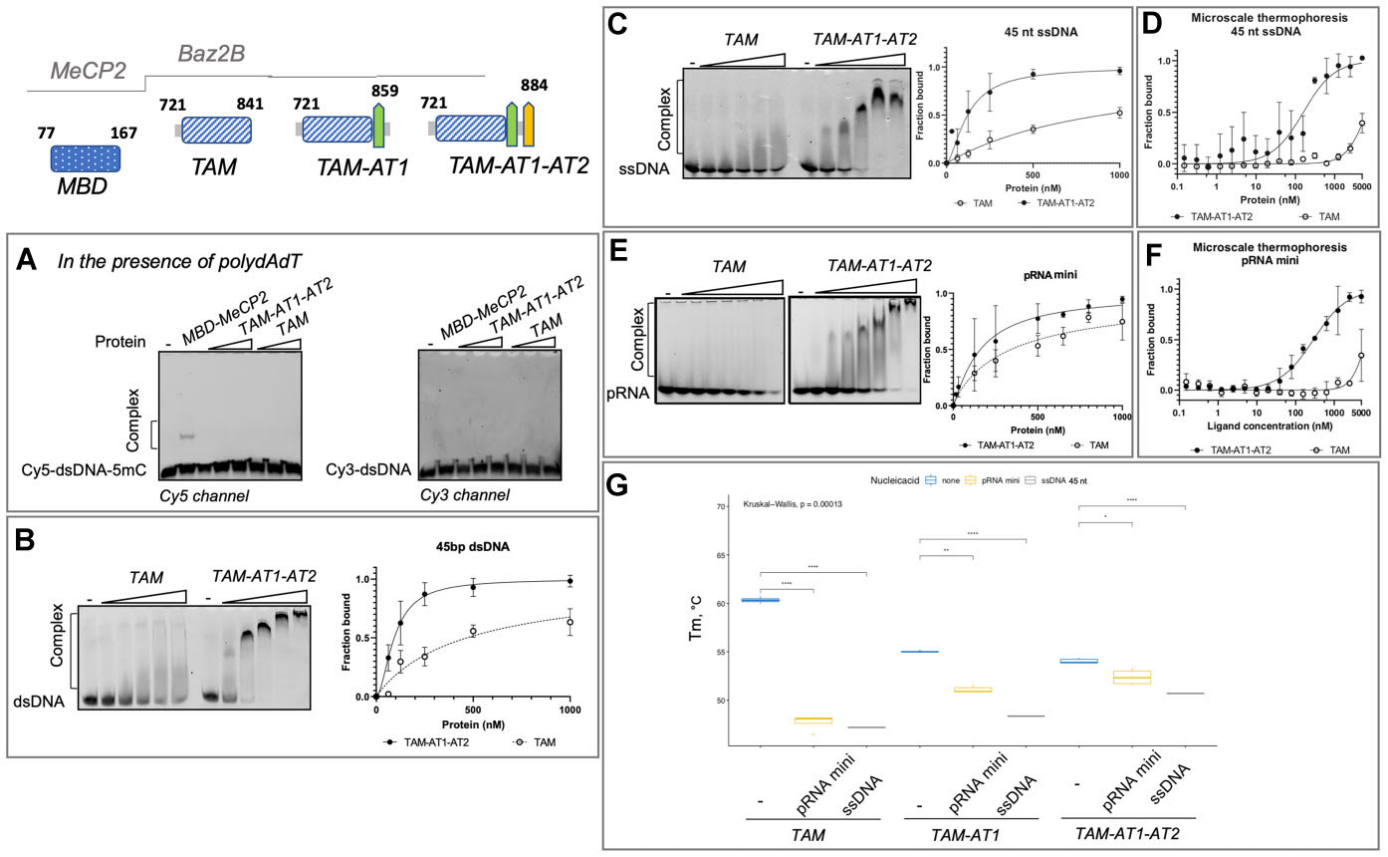
Binding of TAM^Baz2B^ domain with auxiliary AT-hooks to DNA and RNA. (**A**) MBD^MeCP2^, but not TAM^Baz2B^ or TAM-AT1-AT2^Baz2B^ recognizes the 5mC mark on the DNA. In the same reaction mixed 1:1 ratio of the Cy5-labelled 5mC DNA and Cy3-labelled unmodified DNA and incubated either with MBD^MeCP2^, TAM^Baz2B^ or TAM-AT1-AT2^Baz2B^ in the presence of competitor poly(dA-dT) DNA. Binding reactions were analysed by EMSA and the fluorescence images are shown. (**B**) Cy5 labelled 45 bp DNA was incubated with increasing concentrations of TAM^Baz2B^ or TAM-AT1-AT2^Baz2B^ and binding reactions were analysed by EMSA. (**C**) Cy5 labelled 45nt ssDNA was incubated with increasing concentrations of TAM^Baz2B^ or TAM-AT1-AT2^Baz2B^ and binding reactions were analysed by EMSA. (**D**) Binding of TAM^Baz2B^ and TAM-AT1-AT2^Baz2B^ domains to Cy5 labelled 45 nt ssDNA measured by microscale thermophoresis. Error bars represent s.e.m. for each data point, calculated at least from three independent measurements. (**E**) Cy5-labelled promoter RNA (pRNA) was incubated with increasing concentrations of TAM^Baz2B^ or TAM-AT1-AT2^Baz2B^ and binding reactions were analysed by EMSA. (**F**) Microscale thermophoresis measuring binding of TAM^Baz2B^ and TAM-AT1-AT2^Baz2B^ domains to Cy5 labelled pRNA. Error bars represent s.e.m. for each data point, calculated at least from three independent measurements. (**G**) NanoDSF measurements of TAM^Baz2B^, TAM-AT1^Baz2B^ and TAM-AT1-AT2^Baz2B^ in the presence and absence of pRNA and ssDNA. The measured Tm values are plotted using R-software. The Post-Hoc pairwise comparison was performed and the significant differences between pairs are indicated by stars: * *P*< 0.05, ** *P*< 0.01, *** *P*< 0.001 and **** *P*< 0.0001.

To further investigate experimentally the nucleic acid binding determinants of Baz2B, we expressed and purified the TAM^Baz2B^ domain alone or with C-terminal extensions (TAM-AT1^Baz2B^ or TAM-AT1-AT2^Baz2B^) as His-fusion proteins (Supplementary Figure S4) and performed EMSAs. The MBD domain of MeCP2, known to recognise methylated DNA, was used as a comparator. EMSA experiments showed that the MBD^MeCP2^ domain exhibited a preference for methylated DNA, whereas TAM^Baz2B^ and TAM–AT1–AT2^Baz2B^ did not show such specificity (Figure 2A). This observation is consistent with recent data showing that TAM^Baz2B^ has similar DNA binding affinity for methylated and unmethylated DNA (47). We conclude that TAM^Baz2A^ and TAM^Baz2B^, together with three other mammalian MBD proteins (MBD3, MBD5 and MBD6), do not specifically bind methylated DNA (12,47,73,74).

To determine the DNA binding affinity of the TAM^Baz2B^ and TAM-AT1-AT2^Baz2B^ domains, we performed EMSAs in the presence of 45 bp of fluorescently labelled DNA. Both domains were found to bind the DNA, but the protein-DNA complexes formed showed different migration patterns in the native gel. Only smearing in the gel was observed when the TAM^Baz2B^ domain was added (Figure 2B). Based on the disappearance of the free probe, we quantified the EMSA results and calculated the DNA binding affinities. The TAM^Baz2B^ and TAM-AT1-AT2^Baz2B^ domains bound to the 45 bp DNA with dissociation constants (*K*_D_) of 467 and 82 nM, respectively (Supplementary Table S4). We conclude that similar to TAM^Baz2A^, TAM^Baz2B^ binds double-stranded DNA (Figure 2B) and the DNA binding affinity is synergistically increased by the presence of auxiliary AT-hooks as revealed by EMSAs.

Since the MBD domain of MeCP2 binds tightly to single-stranded DNA (75), we asked whether this was also true for TAM^Baz2B^. To further investigate the nucleic acid binding properties, we performed EMSAs and the microscale thermophoresis (MST) measurements in the presence of 45 nucleotide fluorescently labelled ssDNA (Figure 2C, D). The results showed that both TAM^Baz2B^ and TAM-AT1-AT2^Baz2B^ were able to bind the ssDNA. TAM-AT1-AT2^Baz2B^ bound to ssDNA with *K*_D_ values of 121 and 160 nM, measured by EMSA and MST, respectively, whereas the TAM domain showed higher dissociation constants (*K*_D_ > 900 nM and > 6500 nM) without reaching saturation (Supplementary Table S5). These results suggest that the TAM^Baz2B^ domain with auxiliary AT-hooks can also bind to ssDNA.

Further experiments were performed to investigate whether the TAM^Baz2B^ domain or the TAM-AT1-AT2^Baz2B^ domain would bind to promoter RNA mini (pRNA mini) similar to TAM^Baz2A^ and TAM-AT1-AT2^Baz2A^ (15,76). Therefore, we performed EMSAs, MST and nano differential scanning fluorimetry (nanoDSF) measurements. EMSAs and MST experiments revealed that TAM-AT1-AT2^Baz2B^ interacted with pRNA^mini^ with *K*_D_ values of 136 nM and 288 nM, respectively, whereas the TAM^Baz2B^ domain alone showed reduced binding affinity with *K*_D_ values of 341 and 6714 nM, respectively (Figure 2E, F and Supplementary Table S6).

To gain further insight into the interaction of TAM^Baz2B^ and TAM-AT1-AT2^Baz2B^ with the nucleic acids, we performed the nanoDSF measurements. The apparent melting temperature (Tm) of TAM-AT1-AT2^Baz2B^ was found to be 5°C lower than that of TAM^Baz2B^ (Figure 2G), indicating that TAM-AT1-AT2^Baz2B^ is less folded, consistent with alphafold2 predictions where the AT1-AT2 region is a flexible loop. The presence of ligand RNA or ssDNA decreased the Tm of the TAM^Baz2B^ domain by >10°C, whereas the Tm of TAM-AT1^Baz2B^ and TAM-AT1-AT2^Baz2B^ decreased to a lesser extent, as shown by nanoDSF measurements. In general, the Tm values of both proteins decreased upon ligand binding, indicating that the TAM^Baz2B^ domain and TAM-AT1-AT2^Baz2B^ interact with RNA and ssDNA. Interestingly, the Tm values on the ligand interaction decreased, indicating that the ligands preferentially bind to a less populated conformational state (e.g. a partially unfolded state not too far from the native state) in solution (77). Thus, the TAM^Baz2B^ and TAM-AT1-AT2^Baz2B^ domains bind nucleic acids similarly to Baz2A, but TAM-AT1-AT2^Baz2A^ binds the pRNA^mini^ with 10-fold higher affinity compared to Baz2B (15).

**Figure 3.**
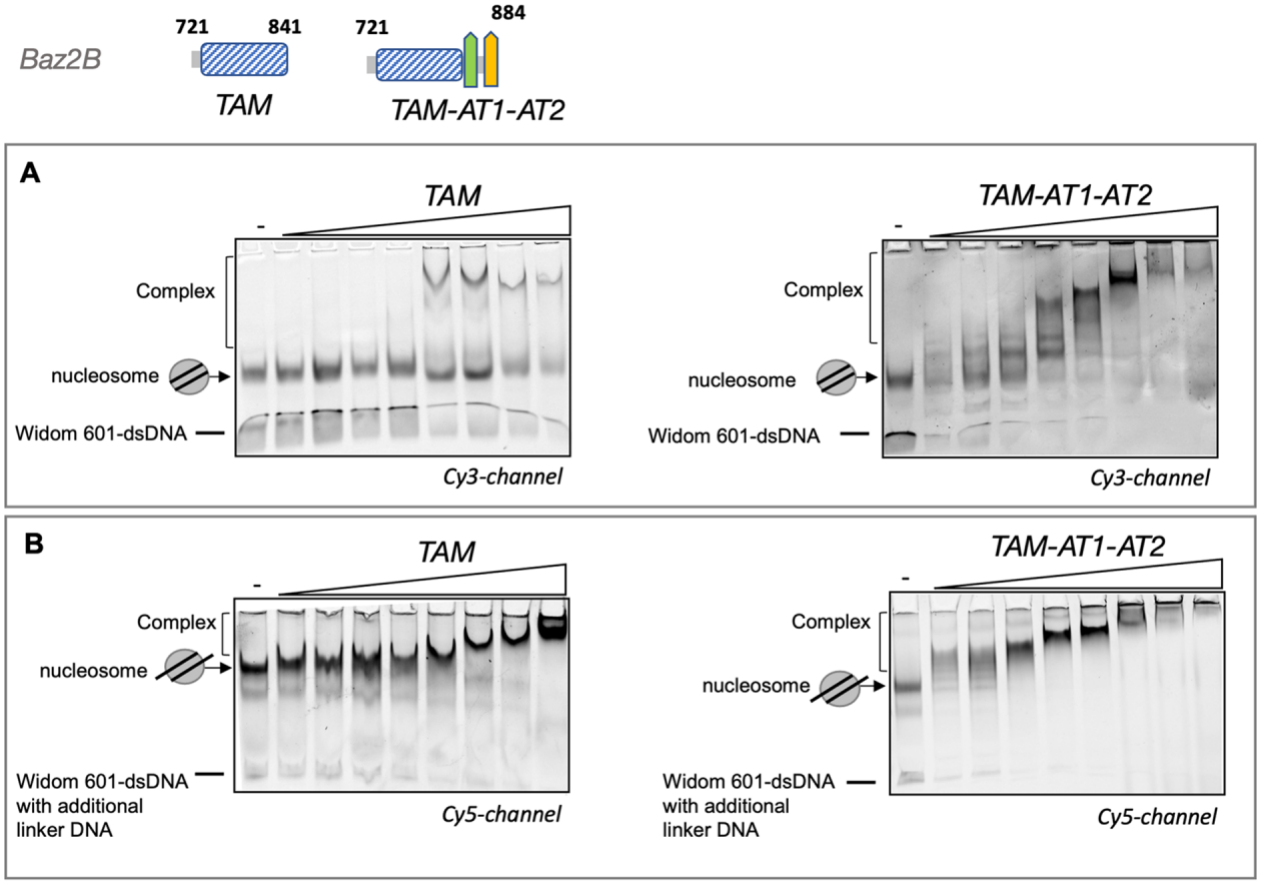
Binding of TAM^Baz2B^ domain with auxiliary AT-hooks to nucleosomes with and without linker DNA. (**A**) The TAM^BazB^ domain (16, 31, 63 125, 250, 500, 1000 and 2000 nM) or TAM-AT1-AT2^BazB^ were incubated with nucleosomes lacking linker DNA. (**B**) Similarly, the TAM^BazB^ domain and TAM-AT1-AT2^BazB^ were incubated with nucleosomes containing 40 bp linker DNA on both sides of the nucleosome. The binding reactions were subsequently analyzed using EMSA and the fluorescence images are shown.

Since cellular DNA is packed into nucleosomes, we next investigated the ability of the TAM^Baz2B^ domain to bind nucleosome-encapsulated DNA. We performed EMSA experiments using calf thymus histones and the Widom 601-DNA sequence packaged into nucleosomes. Different concentrations of TAM or TAM-AT1-AT2 proteins were included in the assays. The EMSA results demonstrated the formation of protein-nucleosome complexes in the presence of TAM proteins, as evidenced by shifted bands on the gel (Figure 3A, B). Interestingly, both nucleosomes with and without linker DNA were bound by TAM^Baz2B^ and TAM-AT1-AT2^Baz2B^ proteins, indicating that the TAM domain can interact with DNA in the nucleosomal context, independent of the linker DNA.

### Baz2B AT-hooks bind DNA

**Figure 4.**
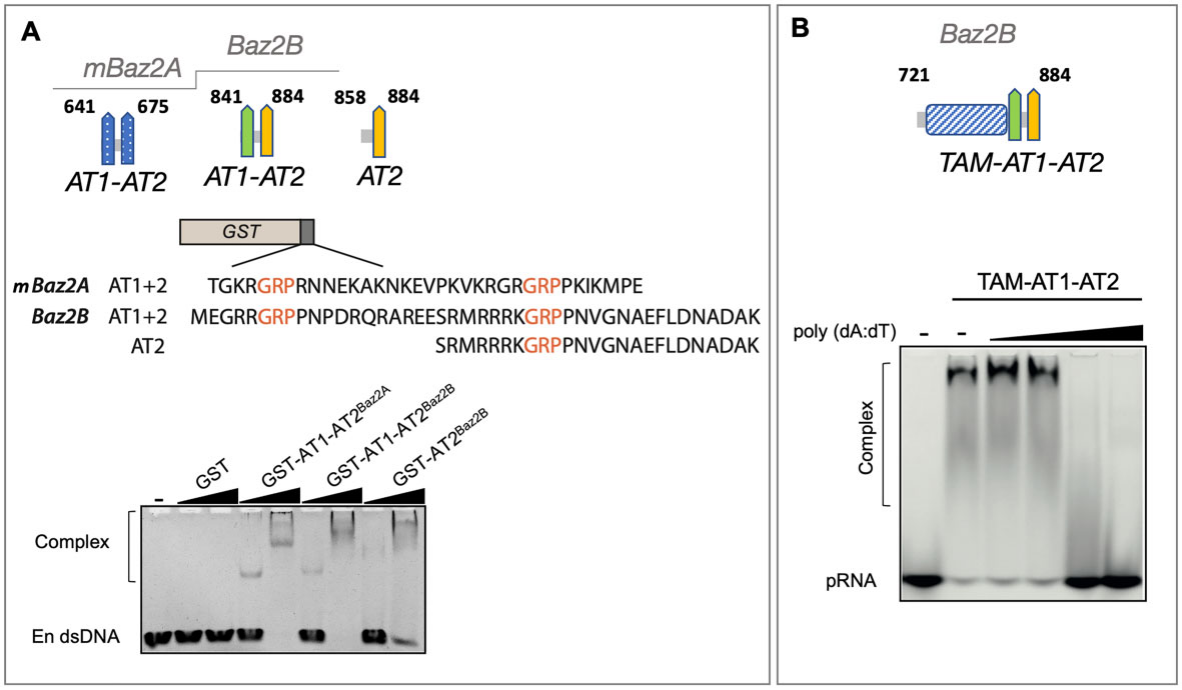
Features of DNA and RNA binding of Baz2B’s TAM domain with auxiliary AT-hooks. (**A**) DNA-binding of Baz2B’s AT-hook motifs. AT-hook motifs were purified as GST fusion proteins. The fused protein sequences are shown. 1.25 μM rDNA enhancer (En dsDNA) was incubated with the 3.6 μM or 14.4 μM negative control GST, positive control GST-AT1-AT2^Baz2A^, GST-AT1-AT2^Baz2B^ or GST-AT2^Baz2B^ and DNA binding ability was monitored by EMSA. The gel was stained with SybrSafe and the obtained image is shown. (**B**) TAM^Baz2B^ domain with the auxiliary AT-hooks binds concurrently with RNA or DNA. Competitive EMSA is shown. 800 nM TAM-AT1-AT2^Baz2B^ was incubated with the 10 nM pRNAmini and an increasing amount of poly(dA:dT) was added (0, 1, 10, 100, 1000 ng). Binding reactions were analyzed by EMSA and the fluorescence image of one out of three representative gels obtained is provided.

The characteristic AT-hook GRP sequence flanked by R/K-rich regions is present at 4 different sites in the Baz2A protein sequence, hence it harbours 4 AT-hooks (AT1, AT2, AT3, and AT4) (21). Interestingly, its paralog Baz2B protein contains two similar sites: AT-hook 1 (AT1) contains the sequence: MEGRRGRPPNPDRQRAREE and AT-hook 2 (AT2) SRMRRRKGRPPNVGNAEFLDNADAK (Figure 4A and Supplementary Figure S1B). To investigate the DNA binding properties of these AT-hooks, we cloned, overexpressed, and purified them as GST-tagged recombinant proteins (Supplementary Figure S5). In the DNA binding assays, we used GST and GST-AT1-AT2^Baz2A^ as negative and positive controls, respectively. Binding reactions were performed on the 34 bp enhancer region of ribosomal DNA (rDNA En) and EMSAs were performed as previously described (21). When higher concentrations of GST-AT1-AT2^Baz2A^, GST-AT1-AT2^Baz2B^ and GST-AT2^Baz2B^ were added to the reaction, slower migrating bands were observed in the gel, indicating complex formation with the DNA (Figure 4A). These results clearly demonstrate that AT-hook1^BazB^ and AT-hook2^Baz2B^ act as auxiliary motifs to the TAM domain of Baz2B, conferring the ability to bind to DNA. The presence of these AT-hooks increases the affinity of the Baz2B protein for nucleic acids. This finding highlights the importance of AT-hooks in mediating DNA-binding interactions within the Baz2B protein.

### Concurrent binding to DNA or RNA by the TAM domain with the auxiliary AT-hooks

In a cellular or physiological environment, Baz2B is constantly exposed to both DNA and RNA. To mimic this simultaneous exposure, we performed studies to explore the binding of TAM-AT1-AT2^Baz2B^ to both types of nucleic acids in single-tube reactions. Competitive EMSAs were performed (Figure 4B) where higher amounts of poly(dA-dT) were added to the RNA and TAM-AT1-AT2 complex. We monitored the increasing amount of free RNA, which migrates fastest in the gel. Interestingly, the migration behaviour of the RNA-TAM-AT1-AT2 complex remained unchanged in the presence of small amounts of DNA, indicating the absence of a ternary RNA-protein-DNA complex. To further validate these findings, we conducted two-colour EMSAs with TAM-AT1-AT2^Baz2B^ and equimolar amounts of RNA and DNA, and the results confirmed that TAM-AT1-AT2^Baz2B^ can bind to either DNA or RNA (Supplementary Figure S6). We conclude that the TAM domain with the auxiliary AT-hooks of Baz2B does not form a ternary complex. This suggests that DNA and RNA probably bind to the same site on TAM-AT1-AT2^Baz2B^ or that the conformation of the formed RNA-TAM-AT1-AT2 complex is not compatible with additional DNA binding.

### Mutational analysis of the TAM^Baz2B^

**Figure 5.**
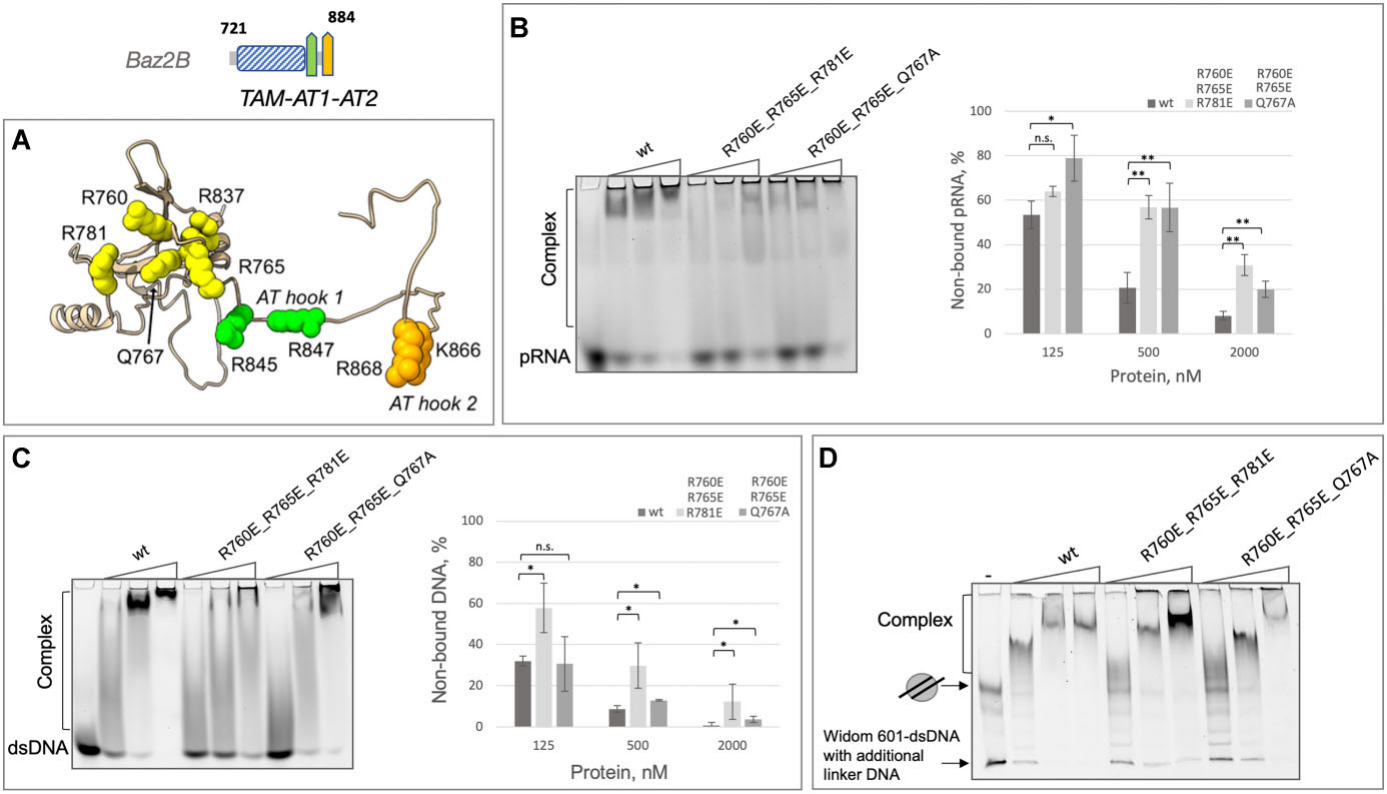
The effect of mutated residues in the TAM-AT1-AT2^Baz2B^ domain. (**A**) Structural representation of mutated amino acid residues in the TAM-AT1-AT2^Baz2B^ domain. Spheres depict the mutated residues, with colours indicating their respective domains: TAM domain (yellow), AT1-hook (green) and AT2-hook (orange). (**B**) Binding of triple mutants of TAM-AT1-AT2^Baz2B^ to pRNA. The wild-type and triple mutants of TAM-AT1-AT2^BazB^ domain were incubated with pRNA at concentrations of 125, 500 and 2000 nM. Binding reactions were analyzed by EMSA, and the fluorescence image of one representative gel is shown. The percentage of protein bound pRNA was determined. Data points represent average values with standard deviation from at least three independent experiments. Significance was determined by one-way ANOVA test, with * indicating *P*< 0.05, ** indicating *P* < 0.01, and n.s. indicating not significant. (**C**) Binding of triple mutants of TAM-AT1-AT2^Baz2B^ to 45 bp dsDNA. The wild-type and triple mutants of TAM-AT1-AT2BazB domain were incubated with 45 bp dsDNA at concentrations of 125, 500 and 2000 nM. Binding reactions were analyzed by EMSA, and the fluorescence image of one representative gel is shown. The percentage of protein bound to dsDNA was determined. Data points represent average values with standard deviation from at least three independent experiments. Significance was determined by on one-way ANOVA test, with * indicating *P*< 0.05 and n.s. indicating not significant. (**D**) Binding of triple mutants of TAM-AT1-AT2^Baz2B^ domain to nucleosomes with linker DNA. The wild-type and triple mutants of TAM-AT1-AT2^BazB^ domain were incubated with nucleosomes at concentrations of 63, 250 and 1000 nM. Binding reactions were analyzed by EMSA, and the fluorescence image of one representative gel is shown. The experiments were conducted at least in triplicate.

The interface between the TAM domain of Baz2A and RNA was previously mapped using NMR and mutational analysis (15). Based on the high sequence identity between the TAM domains and auxiliary AT-hooks of Baz2A and Baz2B (Supplementary Figure S1), we expected that the TAM domains of both proteins would bind the RNA and DNA in a similar manner. To test this, corresponding single-point mutations within the RNA binding interface of Baz2A TAM-AT1-AT2 (K541E, R545E, W546A, R617E) were introduced into the Baz2B TAM-AT1-AT2 domain (R760E, R765E L766A, R837E) (Figure 5A) (15). Q547 from Baz2A was found to be important for RNA interaction in NMR titrations, so Q767A was introduced into TAM-AT1-AT2^Baz2B^. In addition, R781 from Baz2B, which is conserved in all canonical MBDs and critical for MBD1 interaction with methylated DNA, was also investigated.

MD simulations were first performed. Table 2 shows the persistence of nucleic acid contacts with the important TAM-AT1-AT2 residues (as mentioned in the previous subsection) for all five simulated replicates during the 100–300 ns time range (ensuring that the system is far enough away from the initial conformation). The residues, including 845, 847, 866 and 868, which belong to the two AT-hooks, are also shown in Figure 5A and included in the table. Most of the residues that were previously identified as important for nucleic acid binding made significant contacts during the MD simulations, with exception of L766 and R781. Interestingly, in the simulations with dsDNA, the L766 side chain frequently (in almost 60% of the trajectories) made contact with the initially bound AT-hook, with fewer contacts observed in cases where the initial conformations of the nucleic acids were random. Contact with R867 did not appear to occur as it is away from the positively charged TAM-AT1-AT2 plane where the nucleic acids predominantly bind. Consequently, the MD simulations could not explain the importance of R867 in nucleic acid binding.

**Table 2.** The percentage of MD simulation snapshots in the 100–300 ns range in which a specified TAM-AT1-AT2 residue makes a contact with the nucleic acid

Residue	TERRA RNA	ssDNA FLP15	rDNA En (unconstrained)	rDNA En (bound AT1 hook)
R760	47.8	47.9	55.9	50.0
R765	61.0	56.0	70.3	99.7
L766	14.5	17.7	10.7	59.8
Q767	70.0	50.0	44.3	95.5
R781	47.1	87.8	56.7	18.2
R837	0.0	0.0	0.1	0.0
R845 (AT-hook1)	95.4	58.3	73.5	100.0
R847 (AT-hook1)	55.7	36.3	68.8	100.0
K866 (AT-hook2)	78.5	45.9	87.9	78.9
R868 (AT-hook2)	79.2	72.5	78.3	66.5

The snapshots for five replicas in the 100–300 ns range were taken every 0.2 ns.

We then experimentally tested the effect of single point mutations in TAM-AT1-AT2^Baz2B^ on DNA and RNA binding. We generated mutants, expressed, and purified the proteins and performed EMSAs (Supplementary Figure S4A, C). Surprisingly, the single point mutants introduced into the RNA binding interface of the TAM-AT1-AT2 domain of Baz2B did not abolish or significantly reduce RNA or DNA binding (Supplementary Figure S7, Supplementary Table S7). This suggests that the Baz2B TAM domain may bind DNA/RNA differently or that nucleic acid binding is mediated more by auxiliary AT-hooks.

However, it was found that triple mutants on the surface of the TAM^Baz2B^ domain were able to reduce nucleic acid binding, although not completely abolish it (Figure 5 B,C and D). The MD simulations (mentioned above) also indicated that AT-hooks are strongly involved in nucleic acid binding, together with most of the residues mentioned in the mutagenesis study. These results suggest that the TAM domain of Baz2B interacts with DNA/RNA in a more complex and nuanced manner, involving both the TAM domain and auxiliary AT-hooks.

### Bromodomain of Brg1, but not from Baz2B, exhibits binding to double-stranded and triplexed DNA

**Figure 6.**
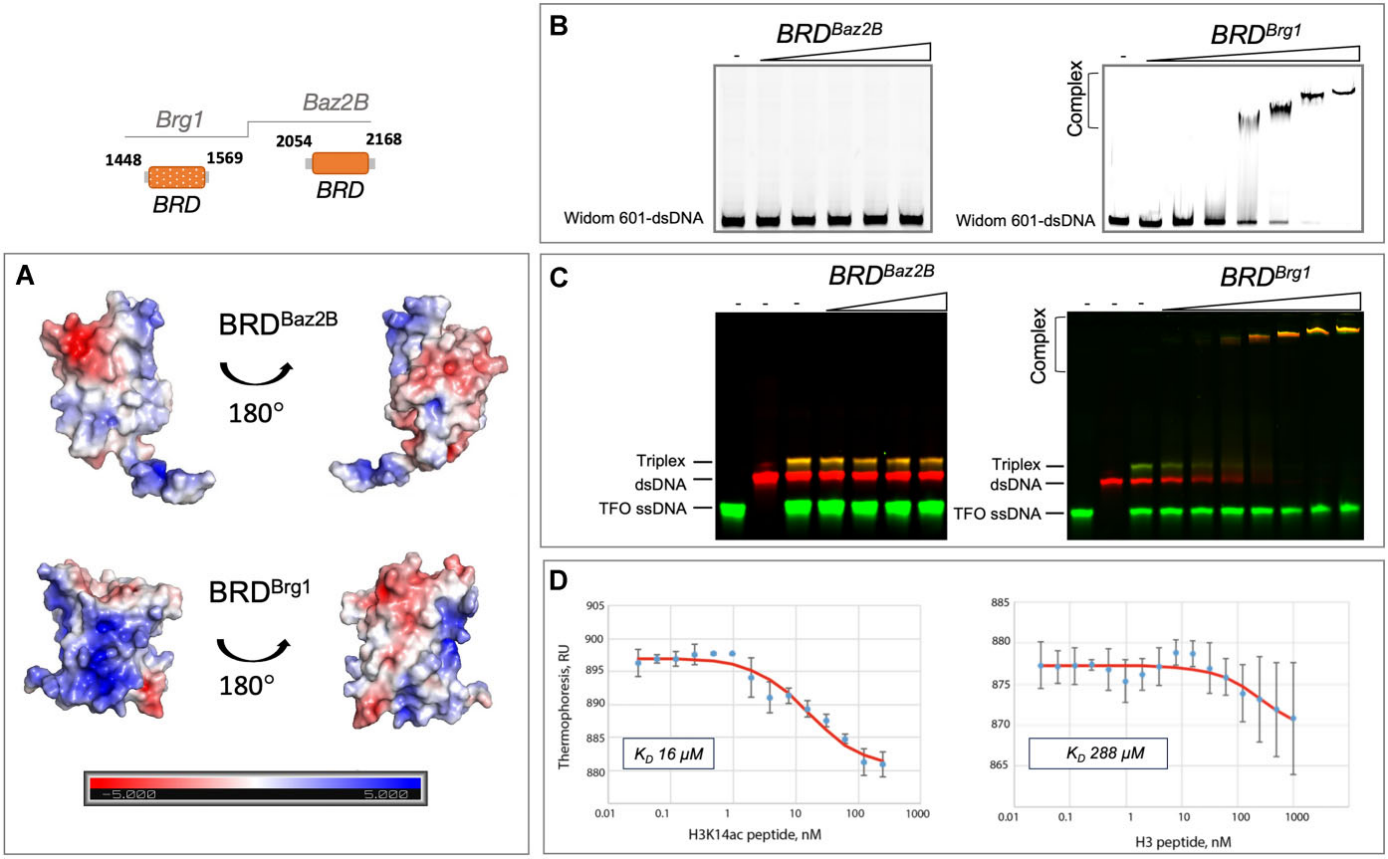
Bromodomain of Baz2B interacts with acetylated histone tails, but not DNA. (**A**) Electrostatic surface of bromodomains from Baz2B and Brg1. APBS-computed surface electrostatics for bromodomains from Baz2B and Brg1, PDB IDs: 4RVR and 2GRC, respectively, were used. (**B**) Various concentrations (0.5, 1, 2, 4, 8, 20, 40 μM) of BRD^Baz2B^ and BRD^Brg1^ (used as positive control), were incubated with 150 nM Cy5-labelled 147 bp Widom 601 DNA and binding was monitored by EMSA. The representative image of the fluorescence scan is shown. (**C**) EMSA analysis incubating the DNA triplex, duplex and TFO (triplex-forming oligonucleotide) with BRD^Baz2B^ (20, 40, 80, 112 μM) and BRD^Brg1^ (0.5, 1, 2, 4, 8, 20, 40 μM). (**D**) BRD^Baz2B^ binding to H3 histone tail and H3K14ac peptides was measured by microscale thermophoresis.

The bromodomains (BRDs) have evolved not only as acetylated histone tail interaction modules but also as DNA-binding domains (28). We therefore investigated whether the bromodomain of Baz2B, which possesses a positively charged surface (Figure 6A), also has nucleic acid binding capabilities. To assess this, we performed EMSAs using recombinantly purified bromodomains of Brg1 and Baz2B. As expected, BRD^Brg1^ bound to 147 base pairs of Widom DNA in our EMSAs, in agreement with previous reports (28) (Figure 6B). In contrast, BRD^Baz2B^ did not show any shifted double-stranded DNA bands under the same and other experimental conditions tested. Considering that the BRD^Brg1^-DNA interaction is driven by its positively charged surface, we hypothesised that the additional negative charge in DNA might increase the binding affinity of BRD^Brg1^ and potentially BRD^Baz2B^. Next, we used a one-pot approach where 29 nucleotides of single-stranded, double-stranded DNA and triplexed DNA were present in a reaction tube and different amounts of bromodomains were added, followed by EMSAs (Figure 6C). As expected, BRD^Brg1^ bound to triplexed and double-stranded DNA but not to the single-stranded DNA. In contrast, BRD^Baz2B^ did not form a complex with any of the DNAs tested.

While it is well established that the acetylated lysine on the histone tail serves as a bona fide substrate for bromodomains, the bromodomain of Baz2B recognises the acetylated lysine of histone H3 (H3K14ac) (22,78). To validate that our purified BRD^Baz2B^ indeed binds to the acetylated H3 tail peptide, we performed microscale thermophoresis (MST) (Figure 6D). As expected, BRD^Baz2B^ showed binding to both the acetylated and unmodified H3K14 tail peptides, with binding affinity constants of *K*_D_= 16 μM and *K*_D_= 288 μM, respectively, consistent with previous ITC and NMR data (79).

In conclusion, our experiments demonstrated that the BRD of Baz2B binds specifically to H3K14ac but not to DNA. Although the BRD carries a positively charged surface, we cannot exclude the possibility that it may contribute to nucleic acid binding when present in the full-length Baz2B protein. Thus, our results show that the DNA-binding properties of bromodomains are not universal and that bromodomains capable of binding double-stranded DNA can also bind triplexed DNA, as demonstrated by the BRD of Brg1, thereby expanding their substrate repertoire.

### Functional consequences of missense mutations found in cancer

Next-generation sequencing has revealed that many chromatin-remodeling proteins are mutated in various cancers. Non-synonymous single nucleotide polymorphisms mapping to the Baz2B bromodomain, obtained from the COSMIC and ICGC data portals (Supplementary Figure S8A, B), were present in various cancer patients, and these cancer patients acquired no more than 500 other mutations. Nine missense mutations in the Baz2B bromodomain were selected for further investigation. Most of these mutations were found in the two terminal αB (L2127V, T2138I, F2139C) and αC (D2145N, A2149V) alpha helices, with some located near the acetyl-lysine binding site and loop regions (K2098N, D2143N) (Supplementary Figure S9). Notably, all mutations affected surface-exposed residues, and certain residues (L2127V, D2145N) were not conserved among the Baz2 proteins, making the functional consequences of these mutations less obvious (Supplementary Figure S10).

To gain deeper insights into the functional impact of cancer-associated mutations in the Baz2B bromodomain, we introduced single-point mutations in the BRD of Baz2B. Recombinant proteins were successfully expressed and purified from *E.coli*, and their conformation in solution was assessed. Circular dichroism (CD) spectra of the wild-type and mutant bromodomain variants confirmed that all bromodomain variants were folded (Supplementary Figure S11). However, the intrinsic fluorescence emission spectra showed changes in the local environment near the tryptophan residues, as evidenced by shifts in emission maximum and/or intensity (Supplementary Figure S12). Next, we measured the thermal stability of the wild-type bromodomain and its mutants using nanoDSF. Strikingly, with the exception of the K2098N and T2138I mutants, all the mutants studied exhibited lower melting temperatures (Tm) compared to the wild-type bromodomain (Table 3), suggesting that most single-point mutations significantly destabilise the native state of the Baz2B bromodomain.

**Table 3.** Impact of mutations found in cancer on Baz2B bromodomain function

			*K*_d_, μM	
Single-point mutation	Cancer type	*T*_m_,°C	H3K14ac	H3
wild-type		54.2 ± 0,05	16	>288
L2073V	pancreatic	**44.8 ± 0.09**	**78**	**7**
K2098N	multiple meyoloma	54.8 ± 0.17	**24**	**8**
F2107V	colon	**38.4 ± 0.16**	**NB**	**NB**
L2127V	breast	**49.9 ± 1.03**	**30**	**1,7**
T2138I	multiple meyoloma	54.8 ± 0.12	94	211
F2139C	endometrial	**53.3 ± 0.10**	20	>798
D2143N	breast invasive carcinoma	54.0 ± 0.42	**133**	**42**
D2145N	stomach adeno carcinoma	**52.1 ± 0.11**	**>338**	**5**
A2149V	uterine	**44.3 ± 0.02**	**NB**	**NB**

The differences between the wild-type and the single-point mutants are highlighted by bold font.

The primary biological function of the Baz2B bromodomain is to recognise and bind to the acetylated histone tail H3K14ac (78). Given that all the bromodomain variants were folded, our next focus was to determine whether these cancer-associated mutants retained their ability to bind to histone tail peptides. To address this question, we used microscale thermophoresis to measure the binding affinities of the bromodomains to unmodified and acetylated at K14 (K14ac) N-terminal H3 histone tail peptides (Table 3). The F2107V and A2149V mutants showed a complete loss of binding to the histone tails, whereas the T2138I and F2139C mutants demonstrated similar binding to the wild-type bromodomain. Remarkably, the L2073V, K2098N, L2127V, D2143N and D2145N bromodomain mutants showed improved binding to the unmodified H3 peptide compared to the H3K14ac peptide. Overall, the results indicate that the mutations identified in cancer patients have different effects on the function of the Baz2B bromodomain. These effects can range from no detectable effect to a complete loss of binding. Interestingly, in some cases the mutations even led to increased binding to the unmodified H3 tail compared to the acetylated form. The mutations are likely to affect interactions with the acetylated histone tails by modulating the activity of BRD, either by altering the stability of the four α-helix bundle or by changing the flexibility of the loops that interact with the histone peptide. However, further investigation is required to determine the effect of these mutations on the full-length protein.

### Baz2B-KO cells exhibit morphological changes and impaired growth

**Figure 7.**
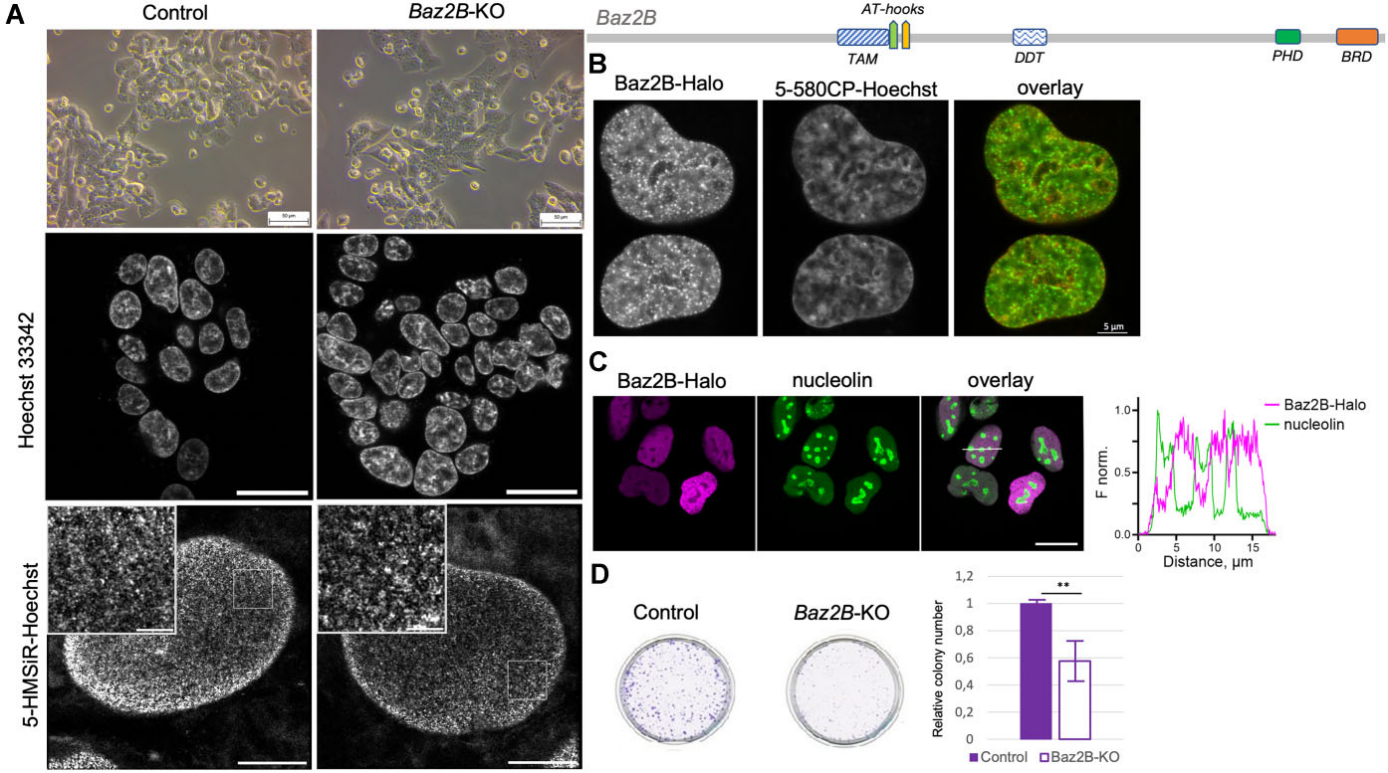
The Baz2B-KO cell line displays different cellular morphology and growth in comparison to the control Hap1 cell line, however, the chromatin structure stays similar. (**A**) Upper, Baz2B knockout changes the morphology of the Hap1 cells. Representative bright-field images of control and Baz2B-KO cells. Baz2B knockout markedly changes the morphology of Hap1 cells, from round to triangular. Middle, nuclei of control and Baz2B-KO cells look very similar when imaged by a confocal microscope. Scale bar – 20 μm. Bottom, no apparent differences in chromatin compaction could be revealed by SMLM. Scale bar – 5 μm, inset – 1 μm. (**B**) Representative image of Baz2B nuclear localization in U2OS cells. (**C**) BAZ2B-Halo protein is excluded from the nucleolus. The U2OS cells were grown for 48 h, then BAZ2B-Halo expression was induced by incubation in the medium with 0.1 μg/ml doxycycline for another 24 h. The cells were stained with 100 nM JF646-Halo substrate in a growth medium for 1 h at 37°C, washed, fixed with 4% PFA and nucleolin was visualized by immunostaining. Left, representative confocal images, scale bar – 20 μm. Right, the profile of the fluorescence signal in the two channels along the indicated line. (**D**) A colony formation assay was performed to analyse the proliferation of control and Baz2B knockout Hap1 cells. Colony formation was significantly reduced in the Baz2B-KO cell line (*n* = 9, quintuplicate technical replicates at three different cell numbers, experiment performed three times). Significance was calculated using a one-way ANOVA test, where ** indicates *P*-value < 0.01.

Depletion of Baz2A has been studied in NIH3T3 and COS cells, where an increase in ribosome production and cell proliferation was observed, whereas in metastatic prostate cancer cell lines, Baz2A paradoxically contributes to cancer cell proliferation and viability (38,80,81). To investigate the cellular role of Baz2B, a Baz2B knockout (Baz2B-KO) was generated by deleting a 16 bp in a coding exon of BAZ2B in the Hap1 cell line using the CRISPR/Cas9 genome editing system (Horizon discovery) (82). Immunoblotting confirmed the absence of Baz2B protein in the Baz2B-KO cell line (Supplementary Figure S13). During cultivation, significant morphological and growth differences were observed between the two cell lines. In particular, the Baz2B-KO cells exhibited a more triangular shape compared to the Hap1 parental control (Figure 7A and Supplementary Figure S14). To investigate whether the absence of Baz2B affects nuclear organisation and leads to the observed morphological changes, the nuclei of both control and Baz2B-KO Hap1 cells were stained with Hoechst33342 dye and visualised by confocal microscopy (Figure 7A, middle panel). However, no obvious differences in the overall nuclear organisation were observed between the two cell lines.

To investigate possible changes in chromatin compaction, we stained cells with the spontaneously blinking DNA probe 5-HMSiR-Hoechst (66) and performed higher resolution Single Molecule Localization microscopy (SMLM) imaging (Figure 7A, lower panel). We identified chromatin nanodomains with a diameter of approximately 90 nm, similar in size to previously described nucleosome clutch domains (83). Notably, there was no significant difference in nanodomain size between control and Baz2B-KO cells, with the maximum Feret diameters of the detected features being 89 ± 6 and 90 ± 3 nm, respectively (Supplementary Figure S15). To exclude the potential for dye competition with Baz2B binding to DNA, EMSAs were conducted (Supplementary Figure S16), and these experiments confirmed the absence of interference. Furthermore, we examined the distribution of histone modifications associated with actively transcribed and repressed chromatin by immunostaining cells with antibodies targeting H3K4ac, H3K27ac and H3K9me2 (Supplementary Figure S17) (84). Once more, our observations indicated no discernible differences between control and Baz2B-KO cells. Additionally, our microscopic analyses demonstrated that Baz2B protein is predominantly localised in the nucleus, but in contrast to Baz2A, is absent from the nucleoli (Figure 7B, C). Taken together, these data suggest that Baz2B is a nuclear protein, but does not induce significant rearrangements of global chromatin organisation in Hap1 cells. To assess the growth differences between the two cell lines, a clonogenic assay was performed (Figure 7D). The results demonstrated a reduced colony-forming capacity of the Baz2B-KO cells compared to the control cell line, suggesting that Baz2B plays a role in cancer cell proliferation in the Hap1 cell line.

**Figure 8.**
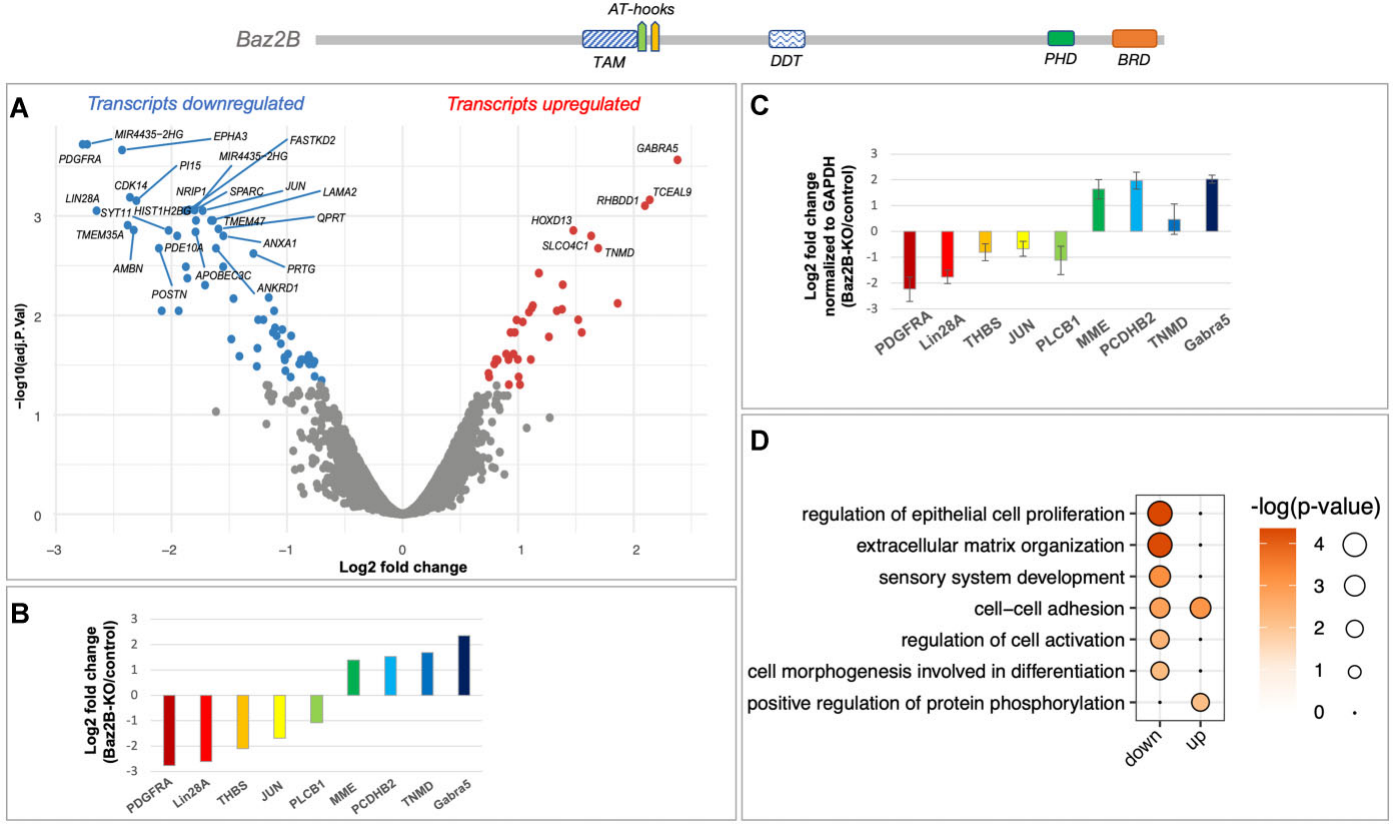
Baz2B regulates gene expression in the Hap1 cell line. (**A**) Volcano plot of differential expression analysis in Baz2B-KO as compared to parental Hap1 control (*n* = 2) cell line. (**B**) Log_2_ fold change of selected genes transcripts from microarray data. (**C**) Validation of fold change expression by quantitative real-time PCR. The other three sets of total RNA were reversibly transcribed into cDNA for qPCR experiments: data were collected by the Rotor-Gene 3000, analyzed using the comparative analysis software module, and presented as relative expression values, using GAPDH for normalization. The mean values and standard deviations are derived from three biological replicates (with at least two technical replicates for each biological replicate). (**D**) Gene-set enrichment analysis.

Chromatin remodelers are known to regulate various DNA-templated processes, including transcription. Transcriptome analysis identified between 1400 and 2000 differentially expressed genes upon loss of the Snf2H catalytic subunit in mouse embryonic stem cells, oocytes or eyes (3,85,86). To investigate the impact of loss of Baz2B, one of the regulatory subunits of Snf2H, on gene expression, transcriptome analysis was performed using the GeneChip® Human Gene 2.0 ST Array. Data analysis revealed 94 differentially expressed genes (FDR < 0.05) in the Baz2B-KO compared to the parental Hap1 control cell line (Figure 8A, Supplementary Table 8). To validate the changes identified on the microarray, real-time quantitative PCR (qPCR) was performed on selected genes (Figure 8B, C). Two sets of genes were chosen, *PDGFRA*, *Lin28A*, *THBS1*, *JUN*, *PLCB1* and *GABRA5*, which were found to be up- and down-regulated, respectively, in support of tumour cell proliferation. The qPCR results confirmed the gene expression changes observed in the microarray analysis. Notably, several oncogenes were downregulated in the Baz2B-KO cells compared to the parental Hap1 control cell line. For example, low GABRA expression is characteristic of hyperproliferative tumours, and higher GABRA5 expression has been shown to inhibit or slow tumour proliferation (87). PDGFRA is elevated in several cancers and promotes tumour proliferation, acting as an oncogene (88). THBS1 promotes tumour cell invasion and growth, and its knockdown inhibits cancer growth (89). JUN is known to promote cancer cell proliferation (90,91) and Lin28A is a known oncogene that promotes cell cycle progression in cancer cells (92). Furthermore, gene-set enrichment analysis (GSEA) revealed seven affected biological pathways in Baz2B-KO cells (Figure 8D). Altogether, these results indicate that Baz2B-KO cells exhibit alterations in the transcriptional landscape during cancer proliferation, while the global chromatin organisation remains similar to that of the parental Hap1 control cell line.

## Discussion

Chromatin remodeling complexes are widely studied and represent an important area of translational medicine due to their increasing relevance in various diseases and ageing. Baz2A and Baz2B are non-catalytic subunits of the chromatin remodeling complexes, but information on the individual domains of Baz2B and its cellular role is limited. In this study, we show that sequences adjacent to the TAM^Baz2B^ domain are the RNA and DNA binding motifs with the AT-hook character and share high sequence identity with the Baz2A AT1- and AT2-hooks, demonstrating that Baz2B, like Baz2A, contains AT-hooks. Our EMSAs and MST analysis show that both the TAM^Baz2B^ domain and the AT-hooks are necessary for robust interaction with nucleic acids. Mutational analysis of TAM-AT1-AT2^Baz2B^ shows that the same point mutations as in Baz2A behave differently in Baz2B and are not sufficient to block the RNA binding. We speculate that TAM^Baz2B^ binds weaker to RNA and the AT-hook interaction is more important, since multiple point mutations are required to affect the nucleic acid interaction. Our MD simulations show that the TAM domain with AT-hook motifs interacts with RNA and DNA, but the interactions with nucleic acids are mostly electrostatic and there are several possibilities. Obviously, in the full-length protein, this flexibility is more constrained by additional protein sequences and domains. We propose a more regulatory role for the TAM domain with the auxiliary AT-hooks within the Baz2B protein. Further studies are required to show whether other nucleic acid binding domains, such as DDT, further increase the nucleic acid binding specificity within Baz2B. Notably, although the TAM-AT1-AT2^Baz2B^ construct has two nucleic acid interaction surfaces (TAM domain and AT1-AT2 hooks), it binds to either DNA or RNA.

Here, we also show that the Baz2B TAM domain with AT-hooks has an affinity for single-stranded DNA. Unusual single-stranded DNA binding properties have also been described for the MBD domain of the MeCP2 protein; remarkably, it binds the single-stranded DNA more tightly than the corresponding duplex DNA (75). The protein could bind the ssDNA or RNA and may be involved in telomere end maintenance, DNA replication, recombination and repair, or simply DNA protection from nucleases. Further studies are required to explore the *in vivo* functional importance of the MBD/TAM domain in ssDNA binding activity.

Another domain within the Baz2B protein that may have nucleic acid binding properties is the bromodomain. In general, bromodomains are known to recognise acetylated histone tails and have emerged as therapeutic targets for the development of specific inhibitors, as their dysfunction has been linked to the development of cancer and other diseases (93–97). In addition to binding acetylated histone tails (H3K14ac), the bromodomain of Brg1, a catalytic subunit of the BAF chromatin remodeling complex, has been shown to bind the DNA (28). The DNA-binding property of the bromodomains does not appear to be a common feature. The bromodomain of Baz2B, although having a positively charged surface, does not bind dsDNA or triplexed DNA as shown in our study. However, although BRD^Baz2B^ does not interact with DNA on its own, we cannot exclude the possibility that BRD may interact with nucleic acids in the context of the Baz2B protein. Furthermore, we show that BRD^Brg1^ binds not only duplex but also triplexed DNA. The triplex structures can form *in vivo* and their presence is not necessarily advantageous as they could promote undesired recombination events and interfere with normal transcriptional controls, therefore another aspect supporting the existence of triplex species could be the presence of proteins that destabilise these structures (98). Superfamily 2 helicases, which include the catalytic subunits of the chromatin remodeling complexes, can displace the third DNA strand from the triplex, leaving double-stranded DNA (99,100). We speculate that the Brg1 bromodomain may recruit the BAF chromatin remodeling complex to DNA triplexes. Whether this is a common feature of bromodomains with DNA-binding activity remains to be shown.

Notably, several point mutations in the Baz2B bromodomain have been identified in human cancers. To investigate the effect of amino acid substitutions on BRD^Baz2B^ structure in solution, thermal stability and histone tail peptide binding, the wild-type and single-point mutant BRD^Baz2B^ were studied. All proteins were folded, but most of them showed altered thermal stability and local conformational changes compared to wild-type BRD^Baz2B^, as revealed by our experiments (Supplementary Figures S11, S12 and Table 3). Binding studies to the H3 tail peptides revealed that the single-point mutants T2138I and F2139C behaved essentially like the wild-type, whereas F2107V and A2149V showed completely abolished binding, most likely due to their massively reduced thermal stability (more than 10°C). Interestingly, other mutants showed reversed binding affinities, with the non-acetylated histone tail binding better than the acetylated tail, i.e. L2073V, K2098N, L2127V, D2143N, and D2145N. To our knowledge, this is the first evidence that cancer might exploit the bromodomains by switching their binding affinity (101).

Taken together, these studies suggest that Baz2B is a nuclear protein that has evolved a TAM domain with auxiliary AT-hooks for RNA, DNA and nucleosome binding and a BRD for acetylated histone tail binding in response to the chromatin environment. These studies will be useful for further characterisation of the MBD domains and the development of Baz2B/Baz2A specific inhibitors.

## Supplementary Material

gkad1096_Supplemental_Files

## Data Availability

The raw and processed microarray data have been deposited in the GEO database under accession number GSE218705.
