# Supplementary material for: Biochemical and cellular insights into the Baz2B protein, a non-catalytic subunit of the chromatin remodeling complex
Source: Nucleic Acids Res. 2023 Nov 24;52(1):337–54. doi: 10.1093/nar/gkad1096 (PMC10783490; doi:10.1093/nar/gkad1096)
Supplement: gkad1096_Supplemental_Files [file gkad1096_supplemental_files.zip › Supplementary data Breindl et al 20230918 revised_corr2.pdf]

Supplementary Information

Biochemical and cellular insights into the Baz2B protein, a non-catalytic subunit of the chromatin remodeling complex

Matthias Breindl[1], Dominika Spitzer[1], Rūta Gerasimaitė[2], Visvaldas Kairys[3], Thomas Schubert[4], Ramona Henfling[1], Uwe Schwartz[5], Gražvydas Lukinavičius[2] and Laura Manelytė[1]*

Supplementary Materials

Table S1. Oligonucleotides used for mutagenesis.

Mutation	***Oligonucleotides used (5'-3')***
TAM domain	
R760E FW	GAAACCCGTATCGAAAACTTCGGTG
R765E FW	CTTCGGTGGCGAACTGCAAGGT
L766A FW	GGTGGCCGTGCGCAAGGTGAG
Q767A FW	TGGCCGTCTGGCGGGTGAGGTG
R781E FW	CAAGAAACTGGAACAGTACCCGG
R837E FW	CGTTATCCCGGAAATTCGTGCGA
MBD FW	AGCAGCAGCACCCTGACCA
LP008 RV[a]	TAGTTATTGCTCAGCGGTGGC
R765E/Q767A RV	CACCTCACCCGCCAGTTCGCCA
Bromodomain	
L2073V FW	CAGCATGATTGTGACCGAAAT
L2073V RV	CACAGCGCCAGATCTTTGCTA
K2098N FW	CGGGCTATAATAAAGTGATTA
K2098N RV	GAACCAGTTTCAGGTTCA
F2107V FW	ACCGATGGATGTTAGCACCAT
F2107V RV	TTTTTAATCACTTTTTTATAGCCCGG
L2127V FW	AACCTTTGCGGTGGATGTGCG
L2127V RV	TCCAGGTTCGGATACTGACCG
T2138I FW	AACTGCGAAATCTTTAACGAA
T2138I RV	ATCAAACACCAGACGCACAT
F2139C FW	TGCGAAACCTGTAACGAAGAT
F2139C RV	GTTATCAAACACCAGACGCA
D2143N FW	TAACGAAGATAATAGCGATAT
D2143N RV	AAGGTTTCGCAGTTATCAA
D2145N FW	AGATGATAGCAATATTGGCCG
D2145N RV	TCGTTAAAGGTTTCGCAGT
A2149V FW	ATTGGCCGTGTCGGTCATAAC
A2149V RV	ATCGCTATCATCTTCGTTAAAGGTT

[a]- The LP008 RV primer was used to generate single-point TAM mutants in all PCR reactions.
FW-Forward
RV-Reverse

TAM-AT1-AT2^{Baz2B} double mutant (R760E/R765E) was generated using pET14b-TAM-AT1-AT2^{Baz2B}-R765E as DNA template and R760E FW and LP008 oligonucleotides.

TAM-AT1-AT2^{Baz2B} triple mutants (R760E/R765E/Q767A and R760E/R765E/R781E) were generated using pET14b-TAM-AT1-AT2^{Baz2B}-R760E/R765E as DNA template and MBD FW and R765E/Q767A or R781E FW and LP008 oligonucleotides.

Table S2. Oligonucleotides that are used in the nucleic acid binding assays.

Name	***Sequence***	***5' label***	***Nucleic acid***	***Purpose***
MeCP2_Cy5_me (sense strand)	TCTGGAA**5mCGG**AATTCTTCTA	Cy5	DNA	To anneal both to produce 5mC containing substrate
MeCP2_me (antisense strand)	TAGAAGAATT**C5mCG**TTCCAGA		DNA	
MeCP2_Cy3 (sense strand)	TCTGGAA**CGG**AATTCTTCTA	Cy3	DNA	To anneal both to produce nonmodified substrate
MeCP2 (antisense strand)	TAGAAGAATT**CCG**TTCCAGA		DNA	
FLP015 (sense strand)	ATCAGTTCTCCGGGTTGTCAGGT CGACCAGTT GTTCCTTTGAGGT	A555	DNA	To prepare 45 bp dsDNA with FLP016/ or use as ssDNA
FLP016 (antisense strand)	ACCTCAAAGGAACAACTGGTCGA CCTGACAAC CCGGAGAACTGAT	None	DNA	
pRNA^{mini}	GGGGGGUCAUUUUGGGGAAAC CCUGUCUCUUUCC	Cy5	RNA	
Telomere RNA	UUAGGGUUAGGGUUAGGGUUAG GGUUAGGGUUAGGGUUAGGG	Cy5	RNA	
rDNA En (sense strand)	TGG ATC TTT TTT TTT TTT TTT CTT TT TCC TCC A		DNA	For preparation of dsDNA
rDNA En (antisense strand)	TGGAGGAAAAAGAAAAAAAAAAAA AAAGATCCA		DNA	
En3_TTS rev TFO#5	GGAGGAAAAAAGAAAAAAAAAAAA AAAGA	Cy3	DNA	For 29 bp triplex formation with 29 bp En3_D duplex
En3_D (sense strand)	TCTTTTTTTTTTTTTTTTCTTTTTTCC TCC	Cy5	DNA	For the generation of 29 bp dsDNA
En3_D (antisense strand)	GGAGGAAAAAAGAAAAAAAAAAAA AAAGA		DNA	

Table S3. Oligonucleotides used in qPCR.

Gene	*Primer (5'-3') Forward*	*Primer (5'-3') Reverse*
JUN	CCTTGAAAGCTCAGAACTCGGAG	TGCTGCGTTAGCATGAGTTGGC
Lin28	CCAGTGGATGTCTTTGTGCACC	GTGACACGGATGGATTCCAGAC
Gabra5	CTGCTCTACACCATGCGCTTGA	GAGCCGTTGGTCCAGACGTAAA
PLCB1	CAACTCACCAAGTCTCCAGTGG	AGTGCCACCATCTGACAACCTG
PDGFRA	GACTTTCGCCAAAGTGGAGGAG	AGCCACCGTGAGTTCAGAACGC
DLK1	CCCCAAAATGGATTCTGCGAGG	GGTTCTCCACAGAGTCCGTGAA
THBS1	GCTGGAAATGTGGTGCTTGTCC	CTCCATTGTGGTTGAAGCAGGC
PCDHB2	ACACTCTGGTGATAAGCACGGC	TCGGAGACCAGCACGGTTATGT
TNMD	GGACTGGTGTTTGGTATCCTGG	CTCCATTGCTGTAGAAAGTGTGC
MME	CTTTAGTGCCCAGCAGTCCAAC	CACCAGTCAACGAGGTCTCCAT
GAPDH	ATGGGGAAGGTGAAGGTCG	GGGGTCATTGATGG CAACAATA

Supplementary methods

Spectroscopic measurements

All spectroscopic measurements were performed at 25°C in 20 mM Tris-HCl pH 7.6 and 200 mM NaCl buffer. The absorbance of the protein solutions at 280 nm was 0.10 AU, corresponding to a protein concentration of 95 μg/ml. Circular dichroism (CD) experiments were performed using a Jasco J-815 spectrophotometer. CD spectra were acquired in the far UV region 190-260 nm at a scan rate of 50 nm/min. Quartz cuvettes with a path length of 1 nm were used. All spectra were collected with a bandwidth of 1 nm and a response time of 2 s, standard sensitivity. All spectra were collected eight times, averaged, and corrected for the buffer spectrum.

Intrinsic fluorescence emission measurements were performed using a Varian Cary Eclipse fluorescence spectrophotometer (Agilent) with a 1.0 cm path length quartz cuvette. Fluorescence emission spectra were recorded from 300 to 400 nm (1 nm sampling interval) with the excitation wavelength set at 295 nm.

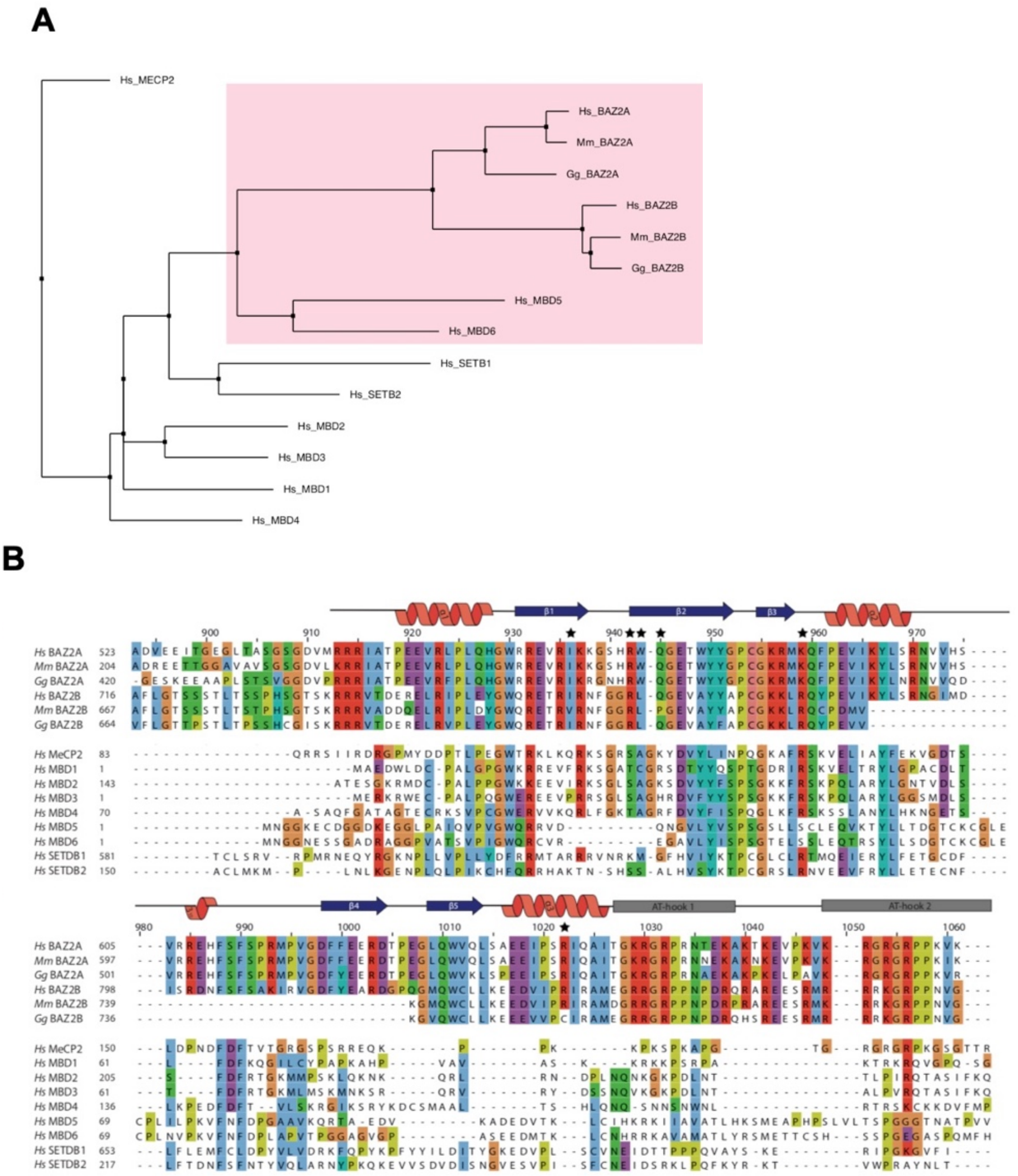

Figure S1. A neighbour joining tree and a multiple sequence alignment of the methyl-binding domains.

(**A**) A neighbour-joining tree of the methyl-binding domains (MBD) of Baz2B and related proteins. The tree is calculated using data from the multiple sequence alignment (B) using the neighbour joining method and the PAM250 substitution matrix in Jalview. (**B**) The alignment was performed using Clustal Omega (1), the figure was created using JalView (2) and edited using Adobe Illustrator. Above the alignment α-helices and β-sheets of the human Baz2B TAM domain in red and blue are indicated, respectively. The prefix indicates the organism, Hs = *Homo sapiens*, Mm = *Mus musculus*, Gg = *Gallus gallus*. The respective amino acid positions of each sequence are shown next to its name. The Uniprot numbers of the sequences used: *Hs* Baz2A-Q9UIF9, *Mm* Baz2A-Q91YE5, *Gg* Baz2A-XP_040510820.1, *Hs* Baz2B-Q9UIF8, *Mm* Baz2B-A2AUY4, *Gg* Baz2B-Q9DE13, MeCP2-P51608, MBD1-Q9UIS9, MBD2-Q9UBB5, MBD3-O95983, MBD4-O95243, MBD5-Q9P267, MBD6-Q96DN6, SETDB1-Q15047, SETDB2-Q96T68. The amino acid exchange mutants, generated in this work, are indicated as black stars.

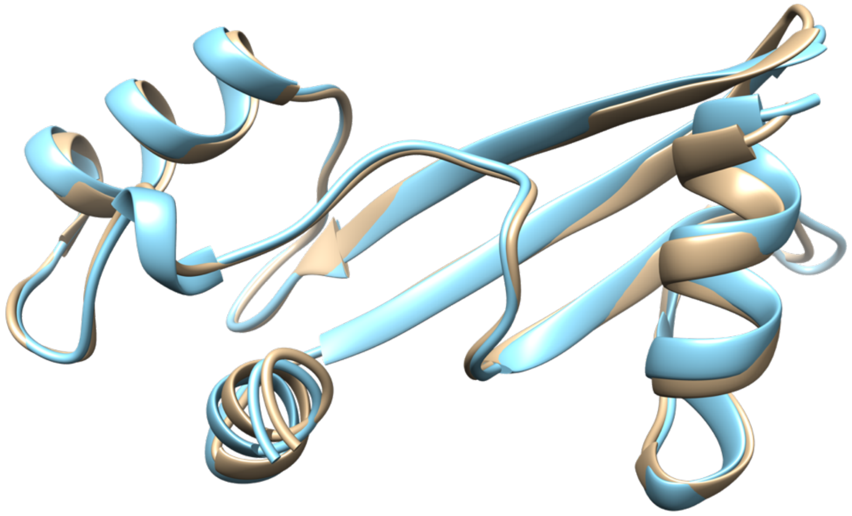

Figure S2. TAM^{Baz2B} domain as predicted by AlphaFold (beige) superposed with X-ray structure (PDB ID: 7WIN, chain A, cyan).

The RMSD between the superposed Cα atoms RMSD is 0.78 Å. The structural superposition was done using MatchMaker tool (3) in Chimera v. 1.16 (4).

Further analysis of the AT hook 1 of Baz2B

The Figure S3 shows a close-up of the residues 845-847 (RGR) shown in Figures 1C and D (left). The AT hook is “wedged” into the minor groove (Figure S3A) and spans the major groove in a relaxed manner (Figure S3B). Figure S3 shows that in the first case the AT hook fits very well to the DNA surface compared to the second case where the Arg side chains are directed by binding to charged phosphate oxygens at the edges of the DNA strands. Analysis using Voronota-JS software showed that the interface areas between the RGR fragment of the protein and DNR are 356 and 249 $Å^2$ respectively, while the unitless interaction scores are −160 and −20, a remarkable difference.

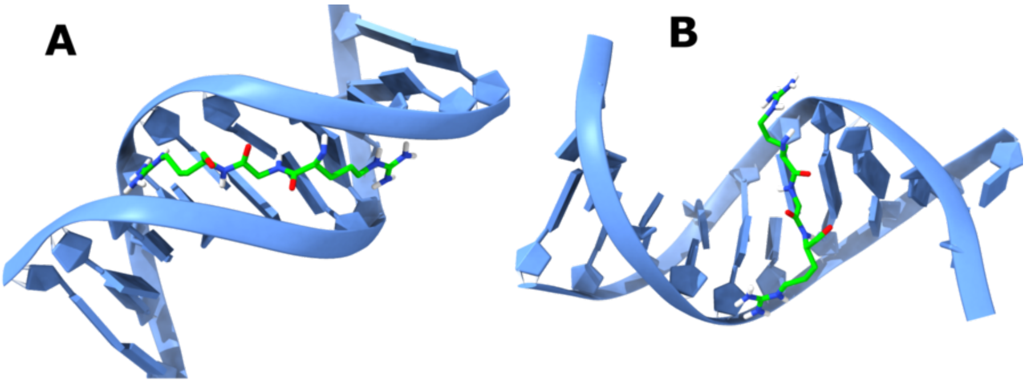

Figure S3. The AT hook RGR (residues 845-847) of Baz2B bound to DNA.

(**A**) and (**B**) correspond to the conformations shown on the left in Figures 1C and 1D, respectively. Clearly, the protein conformation in (**A**) fits the DNA surface much better than (**B**), as reflected in the interface areas and interaction scores (see text). The phosphate backbone is shown as a ribbon.

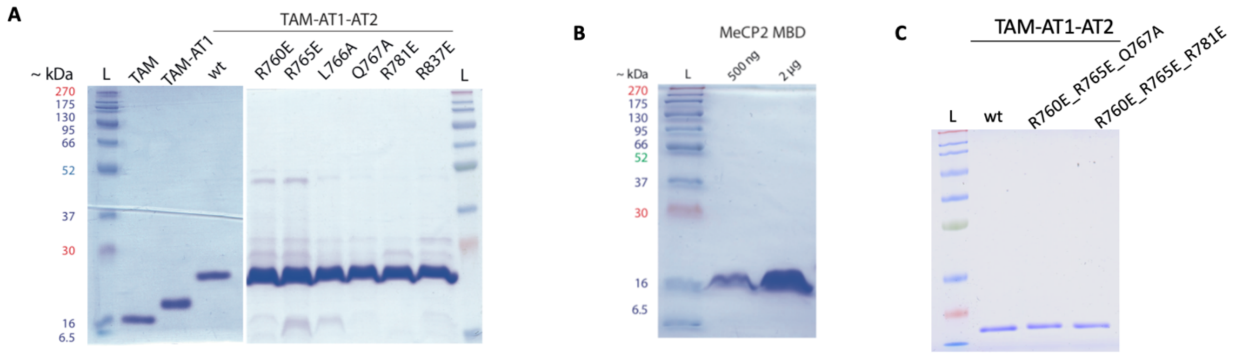

Figure S4. SDS-PAGE analysis of purified domains and mutants used in the study.
(**A**) SDS-PAGE gel displaying purified TAM^{Baz2B}, TAM-AT1^{Baz2B}, TAM-AT1-AT2^{Baz2B}, and single-point mutant proteins. (**B**) SDS-PAGE gel illustrating purified MBD^{MeCP2} protein. (**C**) SDS-PAGE gel exhibiting purified triple-mutants in TAM-AT1-AT2^{Baz2B}. The proteins were resolved on a 15% Tris-glycine gel. The molecular weight standards are indicated in lane L (BLUeye Prestained Protein Ladder (GeneDirex)) for (**A,B**), and lane L is a BlueEasy Prestained Protein Marker (NIPPON Genetics EUROPE) in (**C**).

Supplementary Table S4. Analysis of TAM^{Baz2B} and TAM-AT1-AT2^{Baz2B} binding affinity to 45 bp DNA.

	**EMSA**	
**45 bp DNA with**	**TAM**	**TAM-AT1-AT2**
Specific binding with Hill slope<br>Best-fit values<br>Bmax	=1.000	=1.000
h	0.9711	1.375
K_D (nM)	**467.7**	**82.22**
95% CI (profile likelihood)<br>h	0.6494 to 1.372	1.068 to 1.793
K_D (nM)	343.6 to 687.6	67.17 to 97.20
Goodness of Fit<br>Degrees of Freedom	10	53
R^2	0.8991	0.9038

Supplementary Table S5. Analysis of TAM^{Baz2B} and TAM-AT1-AT2^{Baz2B} binding affinity to 45 nt ssDNA.

	EMSA		MST	
**45 nt ssDNA with**	**TAM**	**TAM-AT1-AT2**	**TAM**	**TAM-AT1-AT2**
Specific binding with Hill slope<br>Best-fit values<br>Bmax	=1.000	=1.000	=1.000	=1.000
h	1.019	1.585	1.667	1.098
K_D (nM)	**899.5**	**121.9**	**6569**	**159.8**
95% CI (profile likelihood)<br>h	0.8193 to 1.251	1.211 to 2.083	1.209 to 2.281	0.7253 to 1.882
K_D (nM)	768.6 to 1099	102.1 to 143.1	5639 to 8444	105.6 to 233.6
Goodness of Fit<br>Degrees of Freedom	13	48	30	44
R^2	0.9579	0.8821	0.8938	0.8181

Supplementary Table S6. Analysis of TAM^{Baz2B} and TAM-AT1-AT2^{Baz2B} binding affinity to RNA.

	EMSA		MST	
**pRNA^{mini} with**	**TAM**	**TAM-AT1-AT2**	**TAM**	**TAM-AT1-AT2**
Specific binding with Hill slope<br>Best-fit values<br>Bmax	=1.000	=1.000	=1.000	=1.000
h	0.906	1.082	2.290	0.9887
K_D (nM)	**341.8**	**136.5**	**6714**	**288**
95% CI (profile likelihood)<br>h	0.6211 to 1.253	0.8011 to 1.463	1.309 to 6.934	0.8546 to 1.152
K_D (nM)	244.3 to 444.5	93.98 to 187.0	5410 to 10998	242.0 to 343.3
Goodness of Fit<br>Degrees of Freedom	25	33	56	46
R^2	0.9152	0.8834	0.4597	0.9585

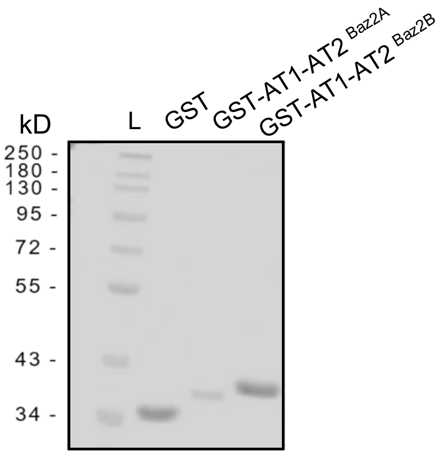

Figure S5. SDS-gel showing the purified GST-fusion proteins: GST alone, GST fused to AT1-AT2^{mBaz2A} and GST fused to AT1-AT2^{Baz2B}.

The purified proteins were loaded onto 15% Tris-glycine SDS gels. L lane is BLUeye Prestained Protein Ladder (GeneDirex).

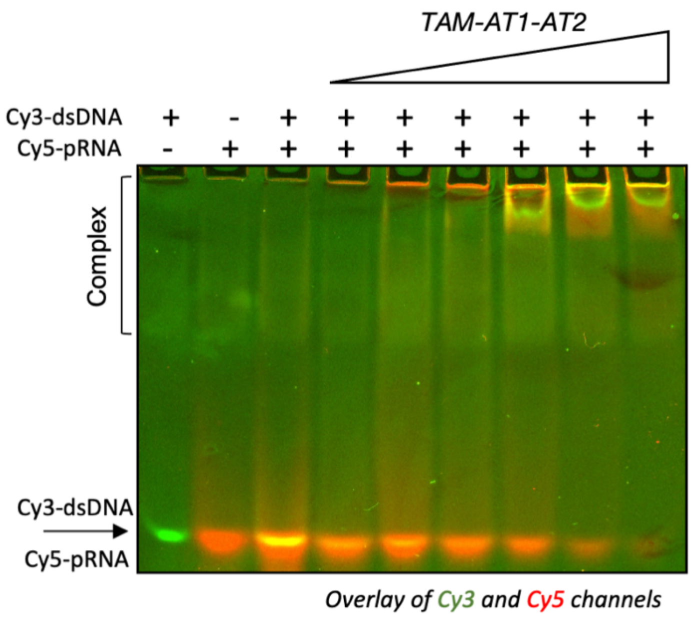

Figure S6. TAM-AT1-AT2^{Baz2B} binds either RNA or DNA.

10 nM Cy5-pRNA and 10 nM Cy3-dsDNA were mixed in EMSA buffer and TAM-AT1-AT2^{Baz2B} at different concentrations (62.5, 125, 250, 500, 1000 and 2000 nM) were incubated and binding reactions were performed as described in Materials and Methods. The overlayed fluorescence image of one out of three representative gels obtained is shown.

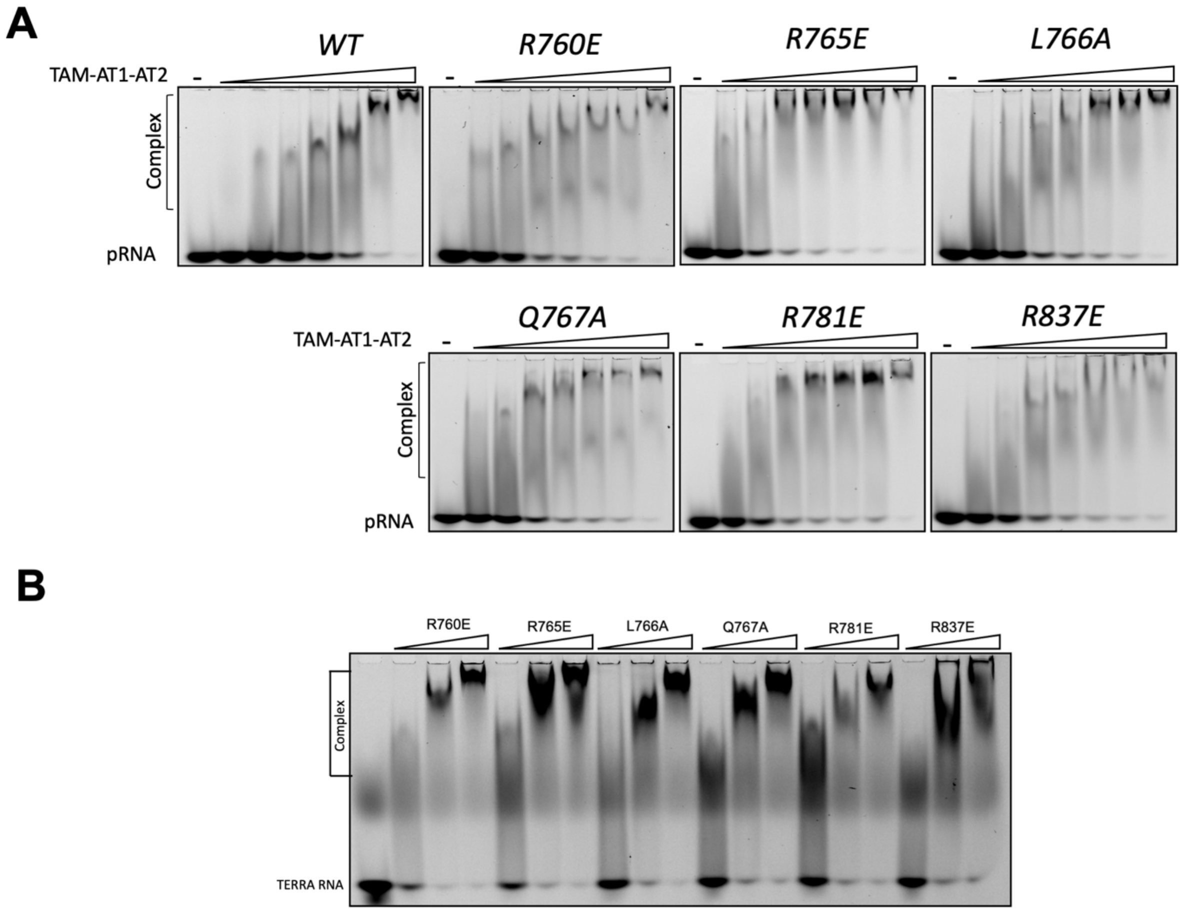

Figure S7. Single point mutations in the TAM-AT1-AT2^{Baz2B} do not abolish RNA binding.

10 nM pRNA mini (**A**) or TERRA-RNA (**B**) and wild-type or single point mutants of TAM-AT1-AT2^{Baz2B} were incubated at different concentrations (125, 500 and 1000 nM) and the binding reactions were analysed by EMSA. The fluorescence image of one out of two representative gels obtained is shown.

Supplementary Table S7. Single point mutations in the TAM-AT1-AT2^{Baz2B} do not abolish ssDNA and dsDNA binding.

TAM-AT1-AT2^{Baz2B}	K_d ± Standard deviation [nM]					
	ssDNA 45 nt			dsDNA 45 nt		
**wt**	**228.1**	±	97.8	**115.9**	±	50.9
**R760E**	**160.8**	±	44.7	**93.6**	±	38.2
**R765E**	**93.1**	±	13.0	**30.8**	±	6.9
**L766A**	**274.6**	±	82.9	**98.1**	±	29.5
**Q767A**	**206.0**	±	81.0	**439.6**	±	256.4
**R781E**	**219.8**	±	109.6	**232.2**	±	101.0
**R837E**	**247.6**	±	84.2	**107.9**	±	9.3

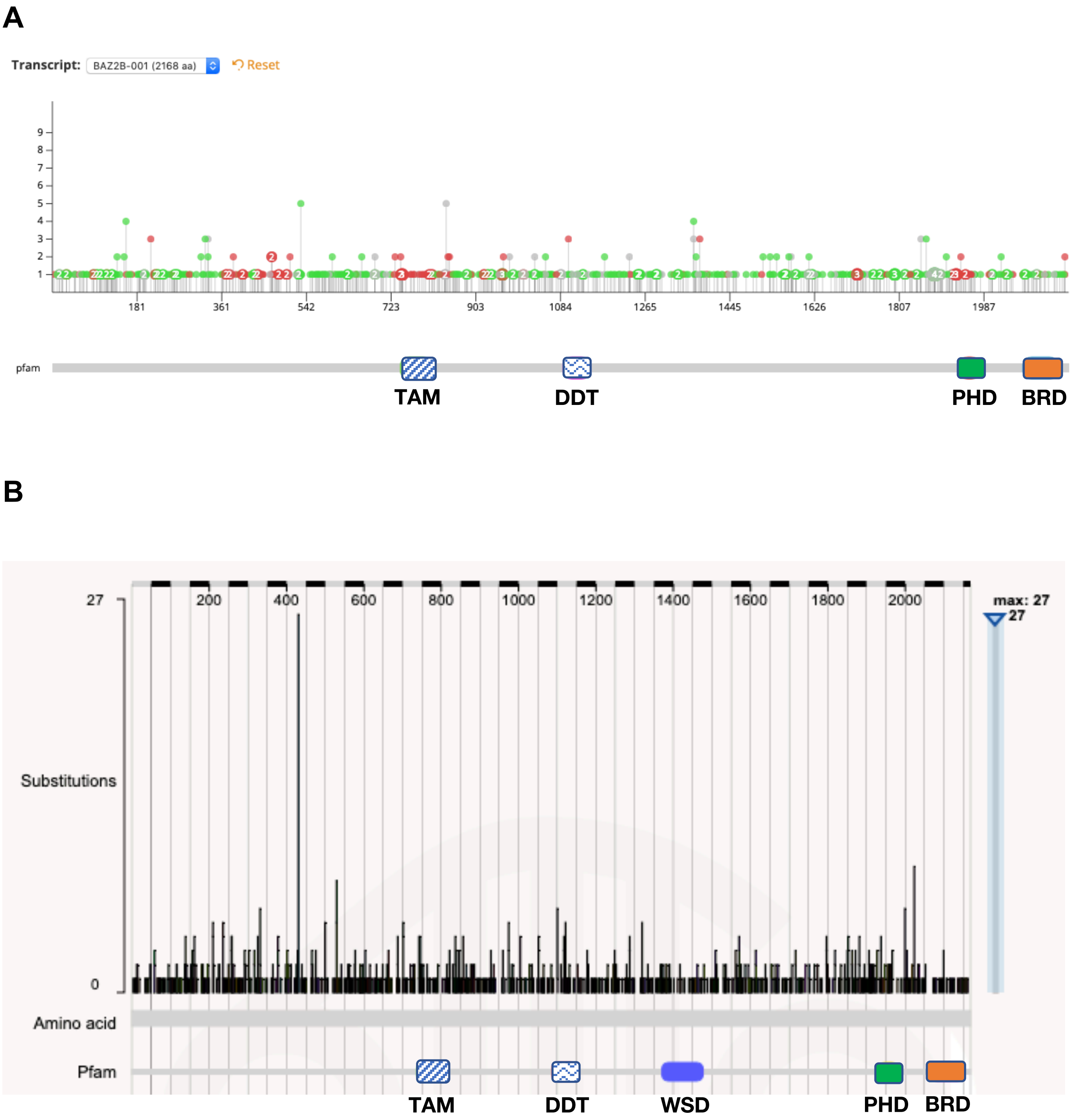

Figure S8. Mutations in the Baz2B protein that occur in cancer.

Snapshots taken in June 2023 from (**A**) ICGC data portal and (**B**) Cosmic database and adapted.

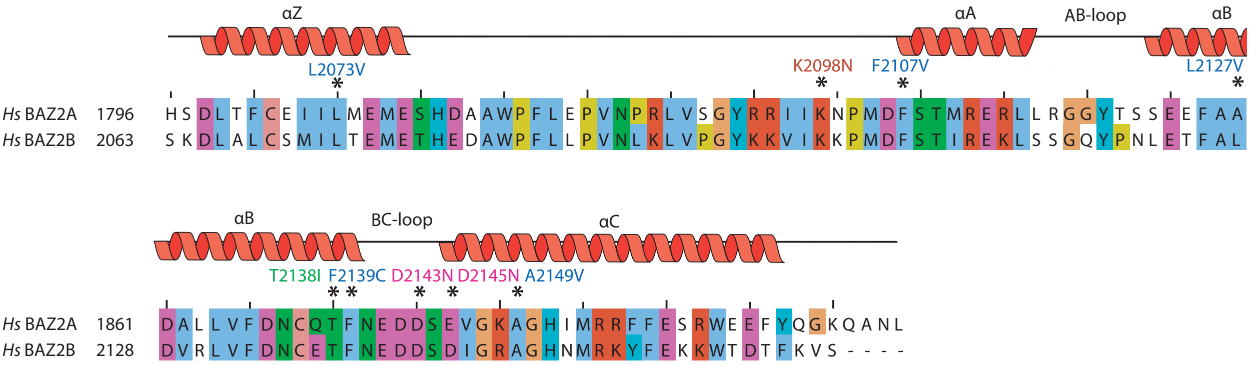

Figure S9. Alignment of the bromodomains of Baz2A and Baz2B.
The alignment was performed using Clustal Omega (1), the figure was created using JalView (2) and edited using Adobe Illustrator. Above the alignment α-helices are marked in red. The prefix indicates the organism, Hs = *Homo sapiens*. The amino acid positions of each sequence are shown next to its name. The Uniprot numbers of the sequences used: *Hs* Baz2A-Q9UIF9 and *Hs* Baz2B-Q9UIF8. The amino acid exchange mutants, generated in this work are indicated by black asterisks.

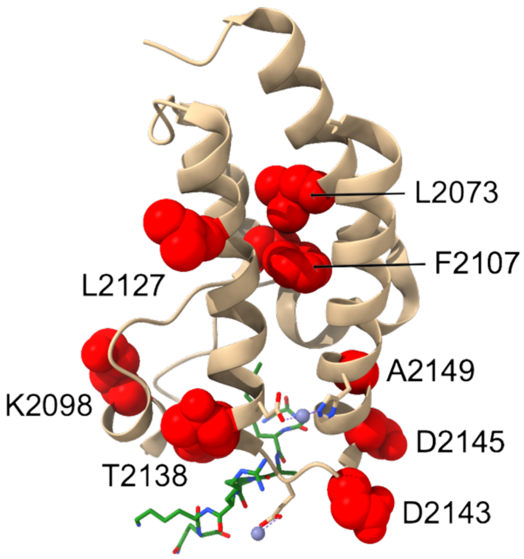

Figure S10. Baz2B bromodomain mutations occurring in cancer.

Location of the mutations in the Baz2B bromodomain. The mutated residues are highlighted in red and are shown as spheres. The bromodomain is shown in light brown and the H3K14ac peptide is shown as sticks with green coloured carbons (PDB ID: 4QC1). The figure was generated using ChimeraX.

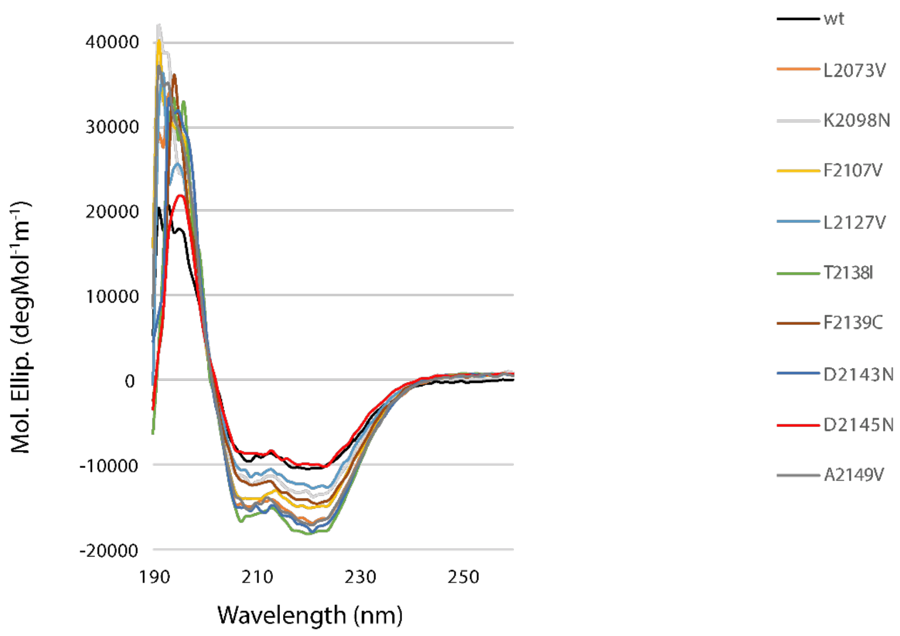

Figure S11. Circular dichroism spectra of BRD^{Baz2B} and the cancer missense mutants.
All spectra were recorded at 95 µg/ml protein in 20 mM Tris-HCl (pH 7.6) and 200 mM NaCl at 25°C.

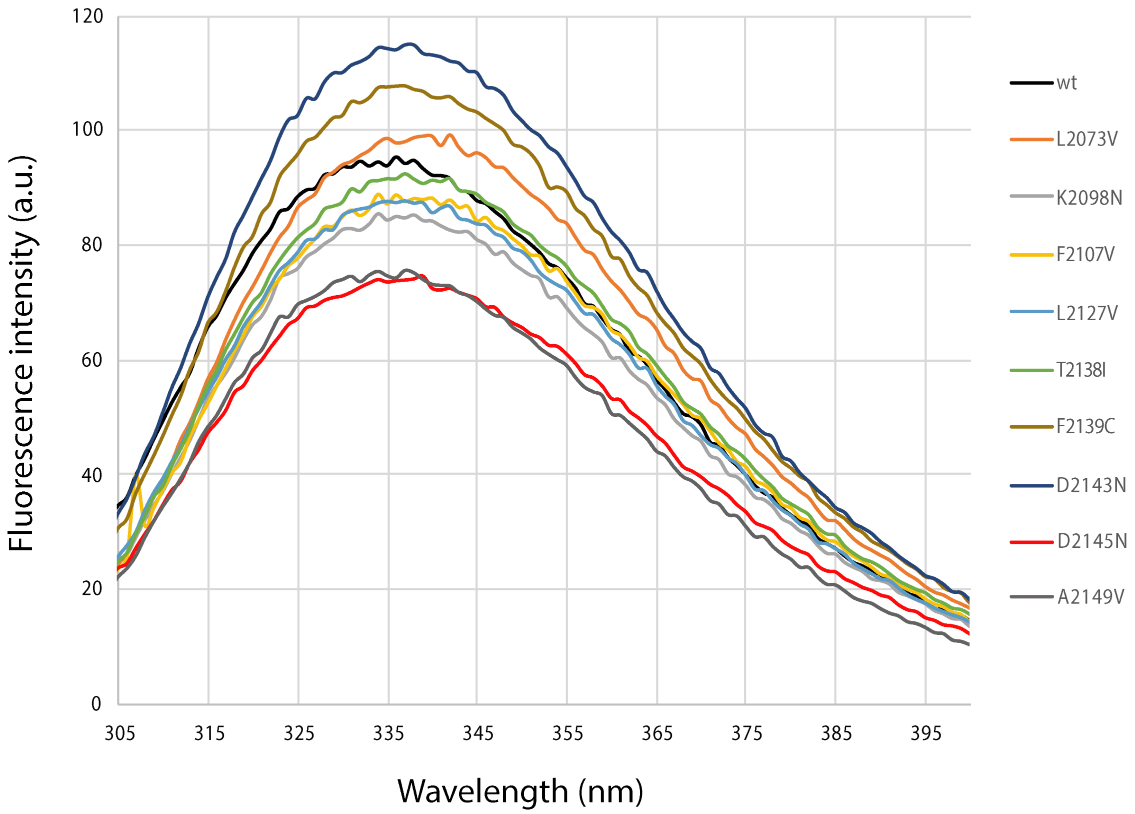

Figure S12. Intrinsic fluorescence emission spectra of BRD^{Baz2B} and mutants.

Intrinsic fluorescence emission spectra were recorded at 20°C in 20 mM Tris-HCl (pH 7.5), 200 mM NaCl and 200 µM DTT. The absorbance at 280 nm was 0.10 AU for all the protein solutions. BRD^{Baz2B} wild-type spectra are shown as black solid lines and mutants are coloured as indicated in the figure.

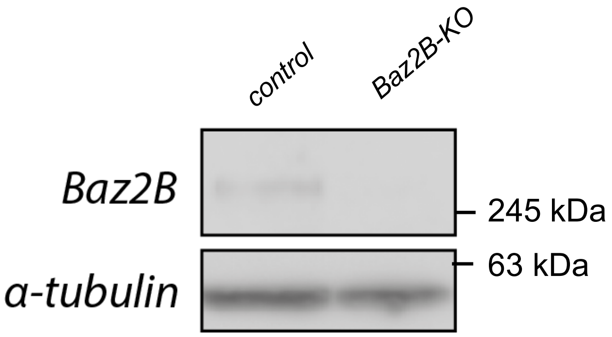

Figure S13. Immunoblot of Baz2B from control and Baz2B-KO Hap1 cell lines.

30 µg of total cellular extract from control or Baz2B-KO Hap1 cells was loaded onto a Bis-Tris 4-12% SDS gel and the gel was electrophoresed in MOPS buffer. Primary rabbit anti-Baz2B (C2) polyclonal antibody (Abiocode) was used to immunoblot the Baz2B protein.

A

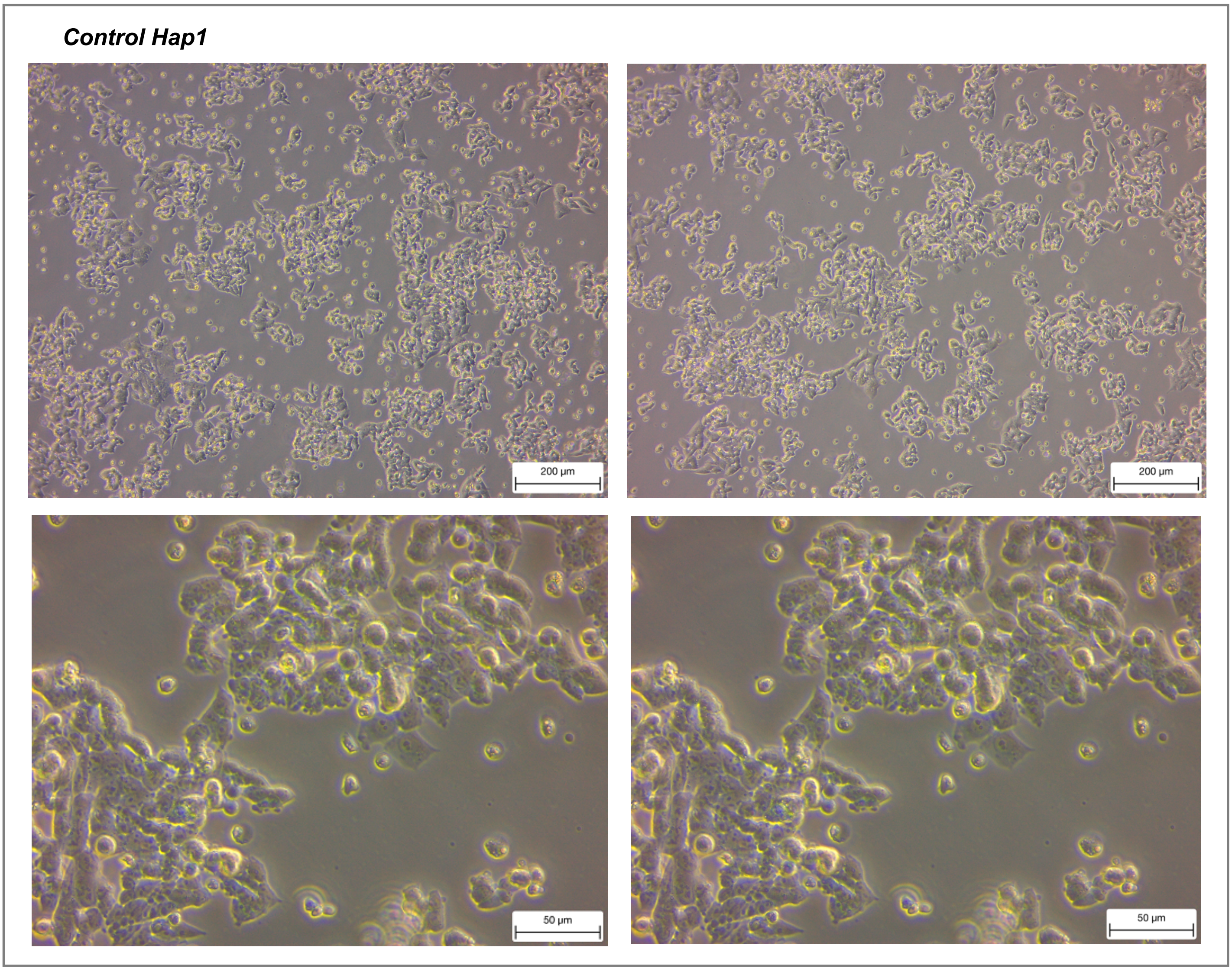

B

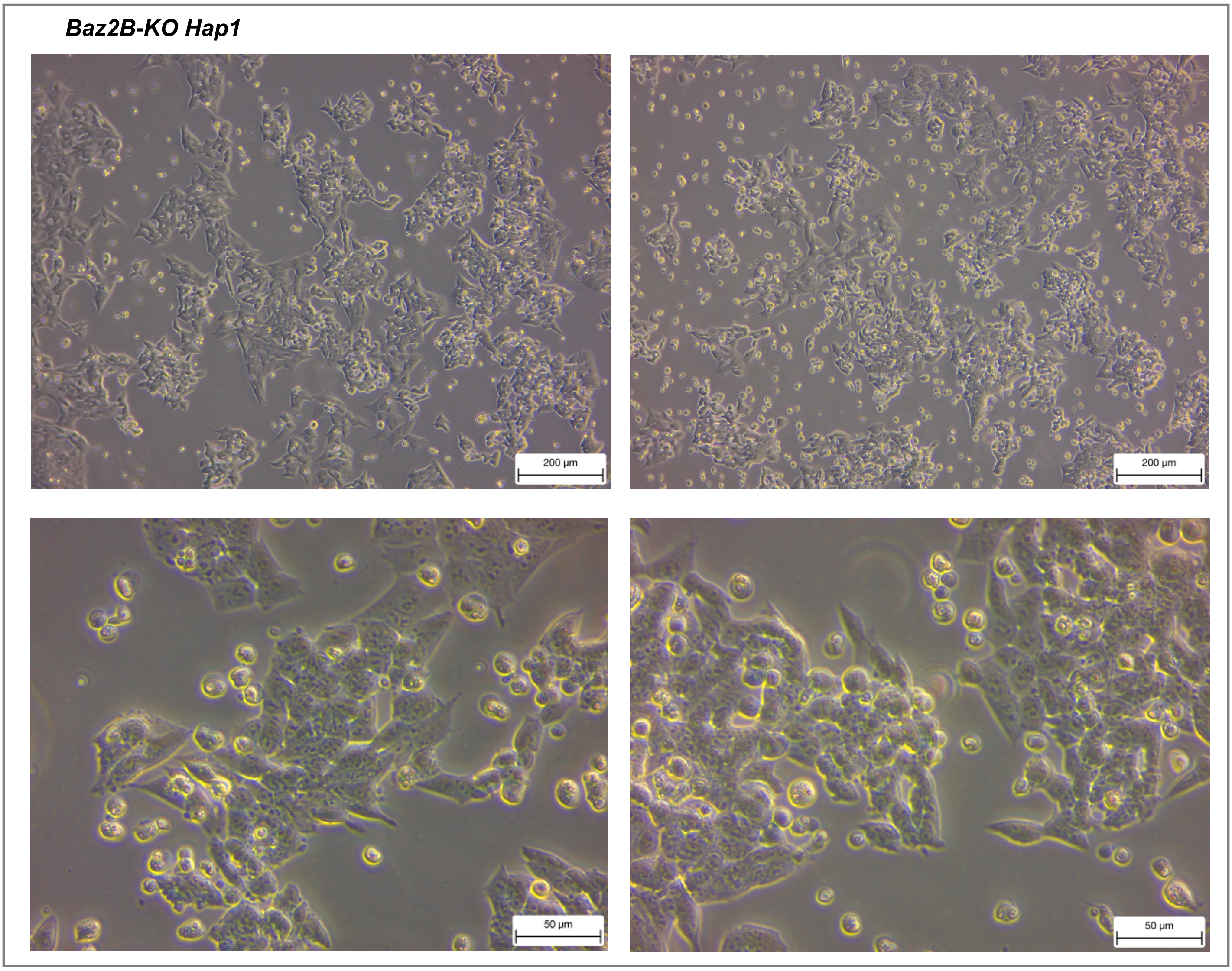

Figure S14. Baz2B knockout alters the morphology of the Hap1 cells.
Representative brightfield images of control and Baz2B KO cells at passage 8. Baz2B knockout clearly changes the morphology of HAP1 cells, from round to triangular.

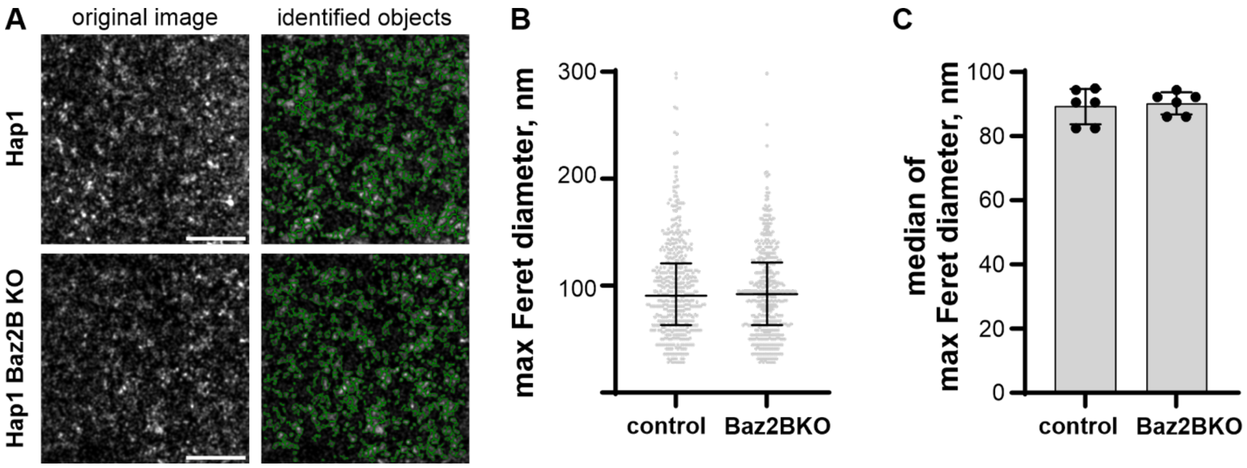

Figure S15. Chromatin nanodomain size is the same in control and Baz2B KO cells.

(**A**) Chromatin nanodomains were identified using the IdentifyPrimaryObjects module of Cell Profiler v.3.1.9 (5), with the typical diameter of objects set to 3-20 pixels. Original SMLM images of chromatin stained with 100 nM 5-HMSiR-Hoechst and overlays with the identified objects are shown. Scale bar – 1 µm. (**B**) Distribution of the nanodomain sizes from the images shown in A. Median and interquartile range are shown. (**C**) Median values of maximum Feret diameter from 6 fields of view. The difference between control and Baz2B KO cells is not significant (p = 0.7110, unpaired two-tailed t-test).

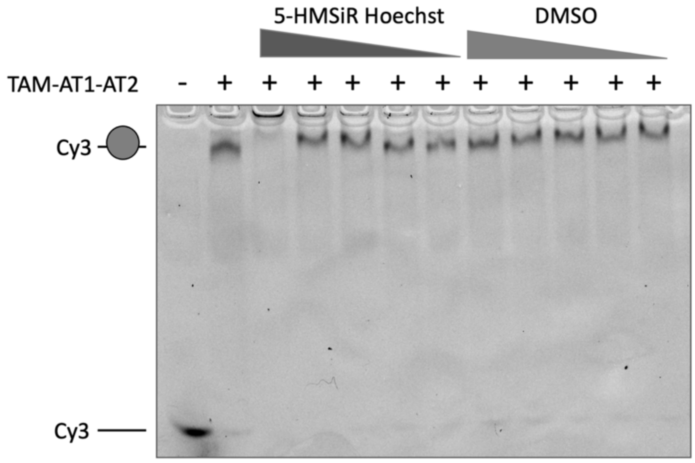

Figure S16. 5-HMSiR Hoechst dye is compatible with TAM-AT1-AT2^{Baz2B} binding to DNA. Representative native gel fluorescence image showing TAM-AT1-AT2^{Baz2B} (500nM) binding to 45 bp dsDNA in the presence of 5-HMSiR-Hoechst (10 μM, 1 μM, 100 nM, 10 nM and 1 nM) and DMSO control (1%, 0.1%, 0.01%, 0.001% and 0.0001%). Note that 100 nM of 5-HMSiR was used in the medium to stain the nucleus.

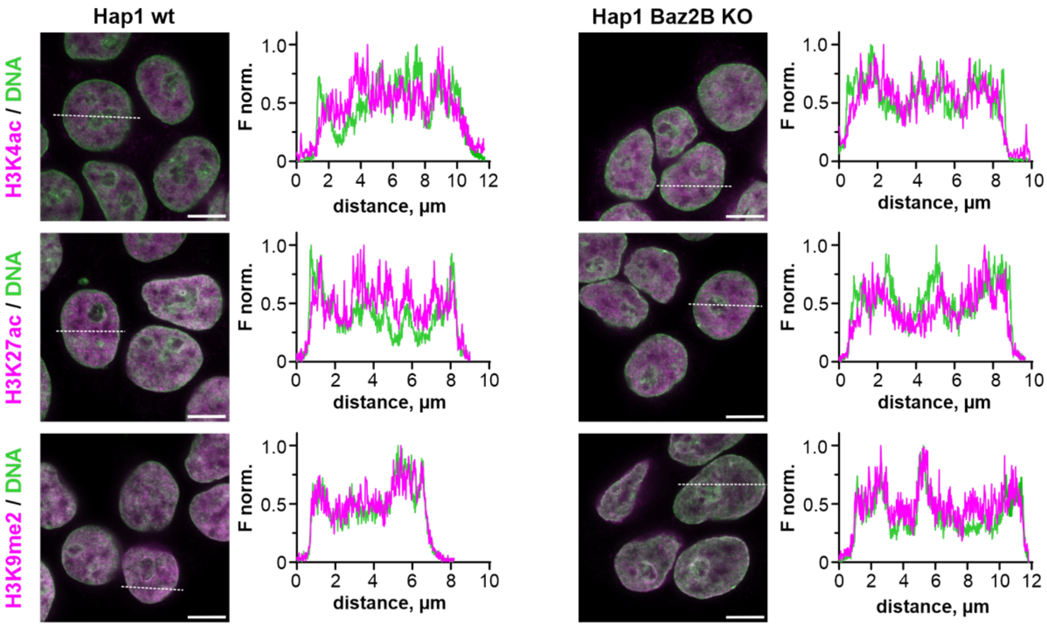

Figure S17. Baz2B KO does not alter the distribution of transcriptionally active and repressed histone marks.

2-colour STED images and profiles along the indicated lines are shown. Scale bar – 5 µm. Note that histone 3 modifications K4ac and K27ac, which are associated with transcriptionally active chromatin, are more abundant in the cell nucleus interior, whereas 5-580CP-Hoechst staining shows distinct peaks in the cell periphery, highlighting A/T-rich heterochromatin associated with the lamina. In contrast, staining for the repressed chromatin mark, H3K9me2, closely follows DNA staining (see profiles next to each image).

The PFA-fixed cells at passage 5 were incubated with antibodies against histone H3 modifications antibodies (Active Motif #39382, #39754 and #39134) at 1:1000 dilution overnight at 4°C, washed three times with PBS and then incubated with a goat anti-rabbit IgG conjugated to Abberior STAR RED for 1 hour. After the last wash, 100 nM of 5-580CP-Hoechst in PBS was added to visualize the DNA, and the samples were imaged without further washing using an Abberior Expert line STED microscope equipped with a UPlanSApo 100×/1.40 oil objective. The following imaging parameters were used: pixel size 20 µm, pixel dwell time 5 µs, pinhole 0.4 AU. Images of antibody and DNA were acquired sequentially, using a 561 nm laser at 20% power and a 640 nm laser at 10% power for excitation and a 775 nm depletion laser at 75% power. The detection windows were 615/20 nm and 685/70nm, respectively.

References

1. Madeira F, Park YM, Lee J, Buso N, Gur T, Madhusoodanan N, et al. The EMBL-EBI search and sequence analysis tools APIs in 2019. Nucleic Acids Res. 2019;47(W1).
2. Waterhouse AM, Procter JB, Martin DMA, Clamp M, Barton GJ. Jalview Version 2-A multiple sequence alignment editor and analysis workbench. Bioinformatics. 2009;25(9).
3. Meng EC, Pettersen EF, Couch GS, Huang CC, Ferrin TE. Tools for integrated sequence-structure analysis with UCSF Chimera. BMC Bioinformatics. 2006;7.
4. Pettersen EF, Goddard TD, Huang CC, Couch GS, Greenblatt DM, Meng EC, et al. UCSF Chimera - A visualization system for exploratory research and analysis. J Comput Chem. 2004;25(13).
5. Mcquin C, Goodman A, Chernyshev V, Kamentsky L, Cimini A, Karhohs KW, et al. Cellprofiler 3.0. PLoS Biol. 2018;
