# Supplementary material for: Biochemical and cellular insights into the Baz2B protein, a non-catalytic subunit of the chromatin remodeling complex
Source: Nucleic Acids Res. 2023 Nov 24;52(1):337–54. doi: 10.1093/nar/gkad1096 (PMC10783490; doi:10.1093/nar/gkad1096)
Supplement: gkad1096_Supplemental_Files [file gkad1096_supplemental_files.zip › Supplementary data S8 Table_legend.docx]

**Supplementary data Table S8**. The microarray data analysis results from the comparative analyses between Baz2B-KO and Hap1 control cells [ log fold change (logFC), average log2 gene expression level across all arrays involved in the comparative analysis (AveExpr), p-value (P.Value)].
